# Overexpression of wild type *RRAS2*, without oncogenic mutations, drives chronic lymphocytic leukemia

**DOI:** 10.1186/s12943-022-01496-x

**Published:** 2022-02-04

**Authors:** Alejandro M. Hortal, Clara L. Oeste, Claudia Cifuentes, Miguel Alcoceba, Isabel Fernández-Pisonero, Laura Clavaín, Rut Tercero, Pilar Mendoza, Verónica Domínguez, Marta García-Flores, Belén Pintado, David Abia, Carmen García-Macías, Almudena Navarro-Bailón, Xosé R. Bustelo, Marcos González, Balbino Alarcón

**Affiliations:** 1 Immune System Development and Function Program, Centro Biología Molecular Severo Ochoa, Consejo Superior de Investigaciones Científicas, Universidad Autónoma de Madrid, 28049 Madrid, Spain; 2 Savana, S.L., Calle Gran Vía 30, 28013 Madrid, Spain; 3 Departamento de Hematología, Hospital Universitario de Salamanca (HUS-IBSAL), CIBERONC (CB16/12/00233) y Centro de Investigación del Cáncer –IBMCC (USAL-CSIC), Salamanca, Spain; 4 Centro de Investigación del Cáncer, Instituto de Biología Molecular y Celular del Cáncer, and Centro de Investigación Biomédica en Red de Cáncer (CIBERONC), CSIC-Universidad de Salamanca, Campus Unamuno s/n, 37007 Salamanca, Spain; 5 Servicio de Transgénesis CBM-CNB, Centro Nacional de Biotecnología Consejo Superior de Investigaciones Científicas, Darwin 3, 28049 Madrid, Spain; 6 Bioinformatics Facility, Centro Biología Molecular Severo Ochoa, Consejo Superior de Investigaciones Científicas, Universidad Autónoma de Madrid, 28049 Madrid, Spain

**Keywords:** RRAS2, RAS proteins, B cells, Lymphoid malignancies, Chronic lymphocytic leukemia, B cell receptor, PI3K pathway

## Abstract

**Background:**

Chronic lymphocytic leukemia (CLL) is the most frequent, and still incurable, form of leukemia in the Western World. It is widely accepted that cancer results from an evolutionary process shaped by the acquisition of driver mutations which confer selective growth advantage to cells that harbor them. Clear examples are missense mutations in classic RAS genes (*KRAS*, *HRAS* and *NRAS*) that underlie the development of approximately 13% of human cancers. Although autonomous B cell antigen receptor (BCR) signaling is involved and mutations in many tumor suppressor genes and oncogenes have been identified, an oncogenic driver gene has not still been identified for CLL.

**Methods:**

Conditional knock-in mice were generated to overexpress wild type *RRAS2* and prove its driver role. RT-qPCR analysis of a human CLL sample cohort was carried out to measure *RRAS2* transcriptional expression. Sanger DNA sequencing was used to identify a SNP in the 3’UTR region of *RRAS2* in human CLL samples. RNAseq of murine CLL was carried out to identify activated pathways, molecular mechanisms and to pinpoint somatic mutations accompanying *RRAS2* overexpression. Flow cytometry was used for phenotypic characterization and shRNA techniques to knockdown *RRAS2* expression in human CLL.

**Results:**

*RRAS2* mRNA is found overexpressed in its wild type form in 82% of the human CLL samples analyzed (*n* = 178, mean and median = 5-fold) as well as in the explored metadata. A single nucleotide polymorphism (rs8570) in the 3’UTR of the *RRAS2* mRNA has been identified in CLL patients, linking higher expression of *RRAS2* with more aggressive disease. Deliberate overexpression of wild type *RRAS2* in mice, but not an oncogenic Q72L mutation in the coding sequence, provokes the development of CLL. Overexpression of wild type *RRAS2* in mice is accompanied by a strong convergent selection of somatic mutations in genes that have been identified in human CLL. R-RAS2 protein is physically bound to the BCR and mediates BCR signals in CLL.

**Conclusions:**

The results indicate that overexpression of wild type *RRAS2* is behind the development of CLL.

**Supplementary Information:**

The online version contains supplementary material available at 10.1186/s12943-022-01496-x.

## Background

B-Cell Chronic Lymphocytic Leukemia (CLL) is the most common cause of leukemia in the Western world and accounts for one-third of new cases of leukemia each year [1, 2]. CLL diagnosis requires the presence of ≥5 × 10^9^/L monoclonal B cells in the peripheral blood with a typical morphology (mature lymphocytes with scarce cytoplasm) and immunophenotype (CD19+/CD5+/CD23+/CD200+) [2]. In cases without lymphadenopathy or organomegaly, a B lymphocyte count below the given level is described as monoclonal B lymphocytosis (MBL). MBL has been found to progress to CLL at a rate of 1–2% per year [3]. CLL has a very heterogeneous clinical course, including patients with a stable form of the disease without treatment requirement, whereas others develop an aggressive form of CLL. Some clinical/biological (male sex, age > 60 years, advanced clinical stage, higher absolute lymphocyte count, high levels of serum β2-microglobulin and LDH) as well as cytogenetic and molecular characteristics (presence of del (17p) and/or TP53 mutations, presence of del(11q), and unmutated IGHV gene have been associated to poor prognosis [3, 4].

The molecular mechanisms underlying CLL have been the subject of extensive research for decades, though their highly dynamic nature and dependence on treatment outcomes often preclude their full elucidation [5]. BCR signaling is crucial in CLL, as evidenced by the dichotomy of disease evolution dependent on BCR mutation status, i.e., IGHV-UM or IGHV-M forms. The involvement of BCR signaling in CLL is also highlighted by current treatments, which focus on inhibiting BCR-associated kinases. Such is the case for ibrutinib, which inhibits Bruton’s tyrosine kinase (BTK), fosfamatinib, inhibiting spleen tyrosine kinase (SYK), or idelalisib, which targets the hematopoietically expressed PI3Kδ [6]. Indeed, PI3Kδ, a downstream player in R-RAS2 signaling, is overexpressed in CLL [7]. Clones of IGHV-M and IGHV-UM B cells are thought to be selected and expand in response to high or low affinity autoantigens, respectively [6]. Notably, BCR signaling in CLL has also been described as cell-autonomous, independent of antigen stimulation and dependent on the heavy chain complementarity-determining region 3 (CDR3) and an internal epitope [8]. This mode of pathogenic signaling points to the importance in CLL of tonic BCR signaling. Repeated BCR stimulation also upregulates CD5 expression, a phenotypic marker for CLL [9].

RAS proteins comprise a family of small guanosine triphosphate hydrolases (GTPases) that include well-known oncogenic players such as K-RAS, H-RAS and N-RAS. The RAS-related subfamily of RAS proteins (R-RAS) are approximately 55–60% identical to their classic counterparts and share factors that mediate their activation-inactivation cycles, i.e. guanine nucleotide exchange factors (GEFs) and GTPase-activating proteins (GAPs). Therefore, they also potentially share activating signals and cascades with classic RAS proteins. Early studies have shown that oncogenic mutations in R-RAS2 have equal or even higher transformation capacities than its classic RAS protein homologs [10, 11]. The overlapping signaling entities and transforming activity of different RAS proteins could suggest redundancy in RAS protein functions, as was found in studies using mice bearing null alleles of RAS family members. However, over the last decades, assays using double or triple knock-out mice and other approaches have clarified the precise involvement of distinct RAS proteins in several processes. Specifically, R-RAS2 has been set forth as an important player in immunological development and homeostasis. R-RAS2 binds to antigen receptors on B and T cells (BCR and TCR, respectively) through their immune Receptor Tyrosine Activation Motifs (ITAMs) and mediates tonic signaling from these key hubs, mainly via PI3K pathways [12]. Downstream of PI3K, R-RAS2 can propagate signals intracellularly through Akt and NFκB [13]. In B cells, we previously showed that R-RAS2, as an effector of the BCR, is required for an efficient germinal center reaction by regulating B cell metabolism [14].

Additionally to its roles in immune homeostasis, R-RAS2 also mediates correct mammary gland development [15], and mutations in *RRAS2* induce breast tumorigenesis and late-stage metastasis [16]. However, a striking early finding was that not only oncogenic mutations, but rather overexpression of the wild-type, unmutated form of *RRAS2* induced breast cancer cell line transformation, as well [17]. Since then, many studies on different types of cancer have reported elevated R-RAS2 levels in human samples, including esophageal tumors [18], oral cancers [19] skin cancers [20] and lymphomas [12]. In spite of frequently finding *RRAS2* mRNA and/or protein overexpressed in human cancers, a causal relationship has not been clearly established. Here, we investigate whether overexpression of wild type *RRAS2*, i.e., without activating mutations, drives the development of malignancies in mice and if there is a correlation with human disease. We find that *RRAS2* overexpression causes CLL in mice and could drive the development of CLL in the majority of human patients.

## Materials and methods

### Mice

**Fig. 1:**
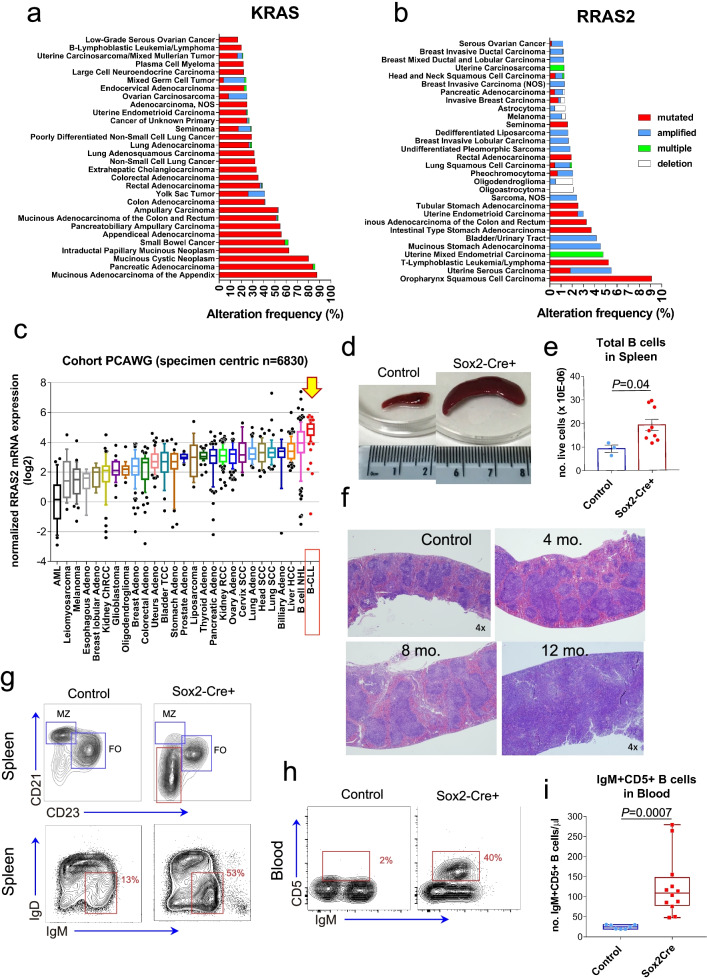
Overexpression of the unmutated RRAS2 leads to a B cell leukemia in mice. **a** & **b** Genomic alterations in the *KRAS and RRAS2* genes according to cBioportal for cancer genomics (http://www.cbioportal.org). Data represent a combined study of 78,278 patients/81072 samples. The X-axis represents alteration frequency of the gene (colors represent the alteration type). The Y-axis represents different cancer types. **c** Relative mRNA expression of RRAS2 in different cancers. Data are represented in log2 scale and were obtained from the Pan-Cancer Analysis of Whole Genomes (PCAWG). **d** Representative images of the relative sizes of spleens from 12 month-old control and Sox2-Cre + mice. **e** Quantification of the total number of B cells per spleen of 6 month-old control and Sox2-Cre + mice. Data shown correspond to triplicate measurements of one control and three Sox2-Cre mice. Unpaired t-test with Welch’s correction. **f** Hematoxylin and eosin images of the spleen structure in an 8-month old control mouse and in 4-, 8- and 12-month-old Sox2-Cre + mice. **g** Two-parameter flow cytometry of the expression of CD21 and CD23, and IgD and IgM, respectively, in B cells in spleens of 8 month-old control and Sox2Cre + mice **h** Left, two-parameter flow cytometry of the expression of CD5 and IgM in B cells of the blood of 8 month-old control and Sox2-Cre + mice. **i,** quantification of the number of CD5 + IgM+ B cells in the blood of 40 wk-old control and Sox2-Cre + mice. Data shown correspond to duplicate measurements of four control and six Sox2-Cre mice. Unpaired t-test with Welch’s correction. In all figures, control mice refer to Rosa26-*RRAS2*^fl/fl^ without Cre recombinase

The Rosa26-*RRAS2*^*fl/fl*^ knock-in mouse line was generated with genOway technologies, inserting by homologous recombination the cassette indicated in Fig. 1A in the Rosa26 locus. This construct is based on the CTV vector (a gift from Klaus Rajewsky; Addgene plasmid # 15912; http://n2t.net/addgene:15912; RRID:Addgene_15,912) [21], and contains the wild-type sequence of human *RRAS2* with an HA-tag under a CAG promoter, followed by EGFP after an IRES sequence and a LoxP-flanked stop codon (Rosa26-*RRAS2*^*fl/fl*^) at the 5′ end of the construct (Fig. S1b and Suppl. File 1). This mouse line was crossed with different Cre recombinase lines, generating conditional overexpression systems by removing the stop codon in specific tissues. We first set out to study systemic *RRAS2* overexpression using Sox2-Cre mice, where Sox2 is an embryonic stem cell transcription factor and therefore induces deletion of the LoxP-flanked sequence in all tissues. Previously described Sox2-Cre and mb1-Cre transgenic mouse lines were gently provided by Dr. César Cobaleda (CBM, Madrid) and Prof. Dr. Michael Reth (University of Freiburg, Germany) [22, 23]. We next generated the Rosa26-*RRAS2*^*fl/fl*^xmb1-Cre B-cell-specific *RRAS2* overexpressing mouse line by crossing Rosa26-*RRAS2*^*fl/fl*^ mice with mb1-Cre mice, which express Cre recombinase specifically in B cells starting at an early precursor phase. Rras2(Q72L)^fl/fl^ xmb1-Cre mice were generated by crossing mb1-Cre mice with Rras2(Q72L)^fl/fl^ mice which have a duplicated and inverted Exon 3 (bearing the Q72L) mutation in addition to the wild type Exon 3. Cre expression leads to swapping Exon 3 and the expression of the mutant Rras2 (Fernandez-Pisonero et al., under revision). Adoptive transfer experiments were performed in a CD45.1 mouse strain. This line was gently provided by Prof. Dr. Carlos Ardavín (CNB, Madrid) [24]. In vivo xenograft tumor growth assay with MEC-1 cells was performed with the immunodeficient *Rag2*^−/−^γc^−/−^ mouse strain [25], purchased from Jackson Laboratories. All mice were maintained under SPF conditions at the animal facility of the Centro de Biología Molecular Severo Ochoa in accordance with national and European guidelines. All the procedures were approved by the ethical committee of the Centro de Biología Molecular Severo Ochoa and were under the Community of Madrid authorization numbers PROEX 384/15 and PROEX 296.7/21.

### Human cells

Human blood samples were obtained from the Center for Blood Transfusions of the ‘Comunidad de Madrid’, where donations were obtained from healthy volunteers after providing their informed consent. Likewise, samples from volunteer CLL patients were obtained from the Hematology Unit of the Salamanca University Hospital after providing written informed consent. Authorization number PI 2019 03217. Fresh human PMBCs were obtained by density centrifugation in a Lymphoprep™ (StemCell technologies) gradient of whole blood for flow cytometry and RT-qPCR analysis.

### Antibodies and reagents

Antibodies used were: anti-mouse IgM-, IgD-, CD21-, CD23-, B220-, CD45R-V450 –biotin –APC, CD11c- (HL3), CD3- (145-2C11), CD4-, CD8a-, Gr1- (RB6-8C5) and NK1.1- (RA3-6B2) –biotin, CD5–PE (53–7.3), CD11b–PerCP-Cy5.5 (M1/70), purified CD16/32 (2,4G2), CD19-PE-Cy7 (1D3), CD45.1 -APC-Cy7 (A20), CD45.2 -APC (104), and AlexaFluor® 647 pBtk (Y223) from BD Pharmingen; anti-human RRAS2 from Abnova, anti-human β-Actin from Sigma-Aldrich (A228), anti-mouse F4/80 –biotin (BM8), from eBioscience; rabbit anti-mouse pERK (T202/Y204) (9101 L), pAKT (S473) (4060 L), pS6 (S240) (5364S), p4EBP1 (T37/46) (236B4), and pBLNK (Y96) from Cell Signalling; pVAV (Y174) (EP510Y) from Abcam, anti-IgM from Jackson Immunoresearch and anti-HA (12CA5) from Sigma.

### Cell preparation

Spleen, lymph nodes and liver from mice of varied ages were homogenized with 40 μm strainers and washed in phosphate-buffered saline (PBS) containing 2% (v/v) fetal bovine serum (FBS). Spleen cells were resuspended for 3 min in AcK buffer (0.15 M NH_4_Cl, 10 mM KHCO_3_, 0.1 mM EDTA, pH 7.2–7.4) to lyse the erythrocytes and washed in PBS 2% FBS. For in vitro cultures, cells were maintained in RPMI with 10% FBS supplemented with 2 mM L-glutamine, 100 U/ml penicillin, 100 U/ml streptomycin, 20 μM β-mercaptoethanol and 10 mM sodium pyruvate.

### Flow cytometry

Mouse single-cell suspensions were incubated with fluorescently labelled antibodies for 30 min at 4 °C after blocking FC receptors using anti-CD16/32 antibody for 20 min at 4 °C. Afterwards, cells were washed in PBS + 2% FBS and data were collected on a FACS Canto II (Becton Dickinson) cytometer. A minimum of 50,000 and a maximum of 200,000 events was acquired in every measurement. Analyses were performed using FlowJo software (TreeStar). Counting of total cells was performed with CountBright™ beads. For Phosflow assays, cells were first stained with extracellular antibodies, and then fixed by adding paraformaldehyde to a final concentration of 2% for 20 min at 4 °C. After two washes, cells were permeabilized for 30 min with 90% methanol on ice. Intracellular labelling was performed at 4 °C O/N with specific phospho-Abs and their corresponding secondary antibodies. For Phosflow assays of the MEC-1 cell line, cells were first serum-starved and treated with specific inhibitors of MAPK extracellular signaling-regulated kinase (ERK) kinase (MEK), U0126 (Promega), phosphatidylinositol 3 kinase (PI3K), wortmannin (Millipore), idelalisib (MedChemExpress) and LY294002 (Sigma), mTOR complex Rapamycin (Millipore), Btk inhibitor Ibrutinib (MedChemExpress) and Src tyrosine kinases family PP2 (Sigma). After 6 h, cells were treated as previously described.

### Adoptive cell transfer

C57BL/6 CD45.1^+^ mice were sublethally irradiated with 0.6 Gy 6 h prior to cell transfer. Splenocytes from Rosa26-*RRAS2*^*fl/fl*^xmb1-Cre mice were prepared as described above and B cells were negatively selected by magnetic bead separa tion using a cocktail of biotinylated antibodies (anti-CD3, anti-CD4, anti-CD8, anti-NK1.1., anti-Gr1, anti-F4/80 and anti-CD11c) followed by incubation with streptavidin-Dynabeads (Invitrogen). Recipient mice were injected intravenously in the tail vein with 5 million purified B cells per mouse. Mice were bled periodically from the facial vein and cells were stained and analyzed by flow cytometry.

### Lentivirus production and generation of MEC-1 RRAS2 knockdown cell line

Lentiviral particles were generated in HEK293T cells cultured in DMEM with L-glutamine 2 mM and 10% fetal bovine serum. Cells were transfected with 1.7 μg pMD2.G, 3.3 μg psPAX2 and 5 μg of lentiviral vector (MISSION® pLKO.1-puro GFP shRNA, Sigma; target sequence 5′-CGTGATGAGTTCCCAATGATT-3′) in jetPEI®. Supernatants were collected and filtered through 0.45 μm filters (Sartorius) 24 and 48 h later. Transduction of MEC-1 cells was performed with Polybrene (Sigma) at 4 μg/ml by centrifugation at 900×g at 32 °C for 70 min without break. Cells were selected in 2.5 μg/ml puromycin 24 h after infection.

### Cell proliferation assay

MEC-1 cells were plated into 24 well plates at the density of 50,000 cells/well. Cells were cultured for 6 days and cell growth was measured in a FACS Canto II using 1μg/ml DAPI (Sigma) as viability dye and CountBright™ Absolute Counting Beads (Invitrogen) for cell count.

### Xenograft tumor growth assay

Ten-week Rag2^−/−^γc^−/−^ male mice were subcutaneously injected in their left flank with 10 × 10^6^ MEC-1 control or MEC-1 *RRAS2* knockdown cells as described in [26], in a mixture with PBS/Matrigel (1:1) (Becton Dickinson). Tumor growth was measured using a caliper, and mice were monitored to avoid unnecessary discomfort according to the ethical guidelines. At the endpoint of the experiment, all mice were sacrificed by CO_2_ inhalation.

### Immunoprecipitation and Western blotting

For Western blotting of whole-cell lysates, GFP^low^ and GFP^high^ populations from Rosa26-*RRAS2*^*fl/fl*^xmb1-Cre mice and splenic B cells from control mice were sorted. Cells were then lysed in Brij96 lysis buffer with protease and phosphatase inhibitors (0.5% Brij96, 140 mM NaCl, 20 mM tris-HCl (pH 7.8), 10 mM iodoacetamide, 1 mM PMSF (phenylmethylsulfonyl fluoride), leupeptin (1 mg/ml), aprotinin (1 mg/ml), 1 mM sodium orthovanadate, and 20 mM sodium fluoride). The samples were then resolved by SDS–polyacrylamide gel electrophoresis (PAGE) and analyzed by Western blotting with anti-human RRas2 from Abnova and anti-human β-Actin from Sigma-Aldrich. For immunoprecipitation, CD19 + CD5+ leukemic cells from spleens of Rosa26-*RRAS2*^*fl/fl*^xmb1-Cre mice were purified by negative selection using a biotin-labelled antibody cocktail and Streptavidin-coupled Dynabeads® (Invitrogen). Plasma membrane proteins of purified CD19 + CD5+ leukemic cells were biotin-labelled using the membrane-impermeable biotinylation reagent Ez-Link SULFO-NHS-LC-Biotin (ThermoFisher). Biotinylated cells were then lysed in Brij96 lysis buffer for 30 min on ice and immunoprecipitation was carried out by incubation of cytosolic fraction with 7 μg of anti-HA (12CA5) previously bound to Protein G Sepharose® beads (Sigma) under continuous rotation at 4 °C overnight. Beads were collected and washed five times with Brij96 lysis buffer and finally resuspended in 20 μL of Laemmli buffer for two-dimensional SDS-PAGE under non-reducing/reducing conditions. Immunoblotting was carried out with streptavidin-peroxidase (Sigma) for identification of total membrane proteins associated with RRAS2, with anti-mouse IgM (Jackson Immunoresearch) and anti-RRAS2 from Abnova.

### Real-time PCR

RNA from peripheral blood mononuclear cells (PBMCs) of CLL patients or Rosa26-*RRAS2*^fl/flx^ mb1Cre sorted mouse splenocytes (GFP^low^ vs. GFP^high^) was isolated using the RNAeasy Plus Mini Kit (QIAGEN). cDNA was synthesized with SuperScript III (Invitrogen) using Oligo-dT primers. Quantitative real-time PCR was performed in triplicate using 100 ng cDNA as template and the reverse transcription reaction with SYBR Green PCR Master Mix and gene-specific primers in an ABI 7300 Real Time PCR System. A set of primers was used to measure mRNA expression of RRAS2 exclusively in human cells (Forward: GCAGGACAAGAAGAGTTTGGA; Reverse: TCATTGGGAACTCATCACGA). A different set of primers was used to measure mRNA expression of combined human (RRAS2) and mouse (Rras2) origin (Forward: GAGTTTGGAGCCATGAGAGA; Reverse: CCTTTACTCTGAGAATCTGTCTTTGA). Both sets of primers expanded exons 3 and 4 in mouse and human mRNA. Obtained cycle threshold (Ct) values were used to calculate mRNA levels relative to 18S rRNA expression using the 2^^(−ΔΔCt) method.

### Sequencing strategy for patients’ samples

cDNA obtained from RNA of patients’ PBMCs as described above was used to sequence the 3’UTR region of RRAS2. A nested PCR was performed for this purpose, using the oligonucleotide with sequence GCAGGACAAGAAGAGTTTGGA, aligning to exon 3 of RRAS2 as FW, and oligonucleotide TGAAGCAGCCTTAGTGTTTCCTT, aligning to RRAS2 3’UTR as RV in the first PCR. For the nested PCR, the product of the first PCR was used as template and the utilized oligonucleotides were TCCATGAACTTGTCCGGGTT, aligning to exon 5 of RRAS2, as FW with the same RV oligonucleotide as in the first PCR. The product of this second PCR was sent to sequence by the Sanger method (Eurofins Genomics), using both FW and RV primers from the second PCR. Both PCR reactions were performed using 30 cycles of 45 s at 95 °C, 45 s at 60 °C and 2 min at 72 °C. All oligonucleotides are indicated in their 5′-3′ orientation.

Alternatively, specific oligonucleotides were used to detect the presence of C or G allele by qPCR using the patients’ cDNA as template. These oligonucleotides were designed as described in [27]. Their sequences can be provided upon request.

### RNA sequencing

Library preparation and sequencing were carried out in ‘Fundación Parque Científico de Madrid’. Briefly, Qiagen RNA Miniprep Kit was used for total RNA extraction following the manufacturer recommendations (including DNase treatment). Once extracted, 100 pg of total RNA from each sample were used as input for library preparation with “NEBNext Single Cell/Low Input RNA Library Prep Kit for Illumina” (New England BioLabs) following the manufacturer recommendations. The so-obtained libraries were validated and quantified in a 2100 Bioanalyzer (Agilent) and an equimolecular pool was made, purified using AMPure XP beads (Beckman Coulter) and titrated by quantitative PCR using the “Kapa-SYBR FAST qPCR kit forLightCycler480” and a reference standard for quantification. The library pool was denatured and seeded on a NextSeq v2.5 flowcell (Illumina) where clusters were formed and sequenced using a “NextSeq 500 High Output kit v2.5” (Illumina) in a 1 × 75 single-read sequencing run on a NextSeq 500 sequencer (Illumina).

### BCR clonality assays

CD19+ CD5+ IgM+ cells were sorted from peripheral blood of Rosa26-RRAS2fl/flx mb1Cre mice using a FACSAria Fusion BSC II sorter and their gDNA was isolated using the QIAamp DNA Mini Kit from QIAGEN. Using this material as template, different PCRs were carried out to detect specific Vh family usage. In all cases, the oligonucleotide GTCTAGATTCTCACAAGAGTCCGATAGACCCTGG aligning to the J3 region was used as reverse with specific oligonucleotides for each of the Vh families as forward. 7183: AAGAASAMCCTGTWCCTGCAATGASC; J558: TCCARCACAGCCTWCATGCARCTCARC; 3609: KCYYTGAAGAGCCRRCTCACAATCTCC; Vh11: GAAGTGCAGCTGTTGGAGACTGGAGAA; Vh12: ATCCGTCAGTCACCTGGGAAACCC. The sequences are presented 5′ to 3′. Degenerated nucleotides are coded as follows: S = C or G; M = A or C; W = A or T; R = A or G; K = T or G; Y = C or T. Oligonucleotides and PCR strategies were designed as described in [28, 29].

### Statistical analysis

Statistical parameters including the exact value of n, the mean +/− S.D. or S.E.M. are described in the Figures and Figure legends. Non-parametric Wilcoxon–Mann–Whitney and parametric Student’s t and One-way ANOVA tests were used as indicated to assess the significance of mean differences. The number of mice to be used for comparison was calculated from preliminary experiments aimed to generate significant data using a two-sided t-test with alpha = 0.05 and a standard deviation of about 0.3. The different deviation of the control and test (leukemic) groups suggested the use of different number of animals of each for the definitive experiments. All data was analyzed using the GraphPad Prism 7 software.

## Results

### RRAS2 is frequently overexpressed in human CLL and its overexpression in wild type form drives the development of leukemia

Approximately 13% of all human cancers have alterations in at least one of the classic *RAS* genes (cBioportal for cancer genomics, www.cbioportal.org). Of those, *KRAS* is the most frequently altered, affecting 10% of patients. Although there are gene amplifications affecting the *KRAS* locus, most of the alterations detected consist of point mutations in the coding sequence (Fig. 1a). Mucinous adenocarcinomas of the appendix and pancreatic adenocarcinomas are the cancers with the highest frequency of missense mutations in KRAS, above 80% (Fig. 1a). In contrast, gene alterations affecting the *RRAS2* locus have been found in less than 1% of all cancers and those alterations involve mostly gene amplifications (Fig. 1b). Squamous cell carcinomas of the oropharynx are the cancers with the highest rate of missense mutations that however barely reach 9% of the tumors (Fig. 1b). In contrast, *RRAS2* is very frequently overexpressed in the wild type form in different types of cancer, being CLL followed by B-cell non-Hodgkin lymphomas the cancers with the highest expression of mRNA for *RRAS2* (Fig. 1c; https://dcc.icgc.org/pcawg). In another previous study, CLL was found as the leukemia with the highest expression of *RRAS2*, peaking at approximately 8-fold more mRNA than blood cells from healthy donors (Fig. S1a, www.oncomine.org [30]).

Given that the wild type form of *RRAS2* has a potent transforming activity of NIH-3 T3 fibroblasts [10], we interrogated if overexpression of wild-type *RRAS2* could cause CLL. To this end, we followed a genetic approach by generating a mouse line with a cassette containing the wild-type sequence of human *RRAS2* under a CAG promoter in the Rosa26 locus, which includes EGFP after an IRES sequence and a LoxP-flanked stop codon (Rosa26-*RRAS2*^*fl/fl*^) at the 5′ end of the construct (Fig. S1b). Crossing this mouse line with different Cre recombinase lines induces removal of the stop codon upon recombination and hence overexpression of R-RAS2 protein in specified tissues. We first set out to study systemic *RRAS2* overexpression using Sox2-Cre mice, where Sox2 is an embryonic stem cell transcription factor and therefore will induce deletion of the LoxP-flanked sequence in all tissues. Rosa26-*RRAS2*^*fl/fl*^xSox2-Cre mice are viable and fertile in hetero- and homozygosity. In control WT mice, *Rras2* mRNA is highly expressed in lymphoid organs (spleen and lymph nodes) compared to other organs like kidneys, skin or liver (Fig. S1c). On top of that, expression of the combined *RRAS2* + *Rras2* mRNA (human and mouse) in Rosa26-*RRAS2*^*fl/fl*^xSox2-Cre mice was found increased in the spleen, lymph nodes, liver, skin and kidneys (Fig. S1c). We did not find evident anomalies in those organs, except in the spleen. Rosa26-*RRAS2*^*fl/fl*^xSox2-Cre mice presented marked splenomegaly (Fig. 1d) that was also manifested in terms of organ weight; spleens of Rosa26-*RRAS2*^*fl/fl*^xSox2-Cre mice were 3-fold larger on average than spleens from control Rosa26-*RRAS2*^*fl/fl*^ littermates (Fig. S1d). Spleen enlargement was paralleled by a net increase in the number of CD19+ B cells, suggesting that splenomegaly was due to B cell lymphocytosis (Fig. 1e). In addition, spleen size increased with age and was concomitant with enlarged follicles and loss of predominant diffuse red pulp areas, as observed by histopathological examination after hematoxylin and eosin staining (Fig. 1f).

The analysis by flow cytometry of marginal zone (MZ) and follicular B cell populations in 8-week-old mice showed the presence of an abnormal and abundant CD19^+^CD21^−^CD23^−^ population in Rosa26-*RRAS2*^*fl/fl*^xSox2-Cre mice, as well as an abundant IgM^+^IgD^−^ population (Fig. 1g). Those populations of B cells could correspond to a transitional T1 population of B cells in their differentiation towards follicular or MZ B cells [31]. However, those cells expressed the CD5 marker, constituting a CD19^+^IgM^+^CD5^+^ population which was abundantly detected in spleens of Rosa26-*RRAS2*^*fl/fl*^xSox2-Cre mice but not in those of control Rosa26-*RRAS2*^*fl/fl*^ mice (Fig. S1e). The abnormal CD19^+^IgM^+^CD5^+^ population was also detected in the bone marrow (Fig. S1f) and was present in high number in the blood (Fig. 1h and i), suggesting that that the lymphoproliferative disease in Rosa26-*RRAS2*^*fl/fl*^xSox2-Cre mice is indeed a B cell leukemia.

**Fig. 2:**
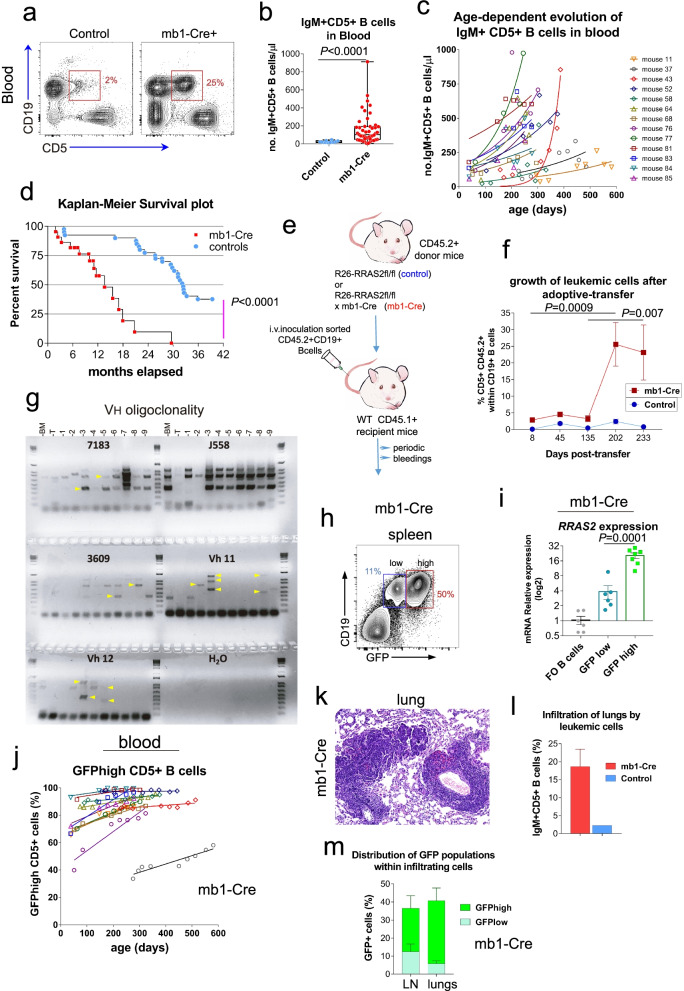
CD5 + IgM+ B cells are oligoclonal and can be transferred to wild type mice. **a** Two-parameter flow cytometry of the expression of CD19 and CD5 in blood cells of 35 wk-old control and Rosa26-*RRAS2*^fl/fl^xmb1-Cre (mb1-Cre+) mice. **b** Quantification of the number of CD5 + IgM+ B cells (CD19+) in the blood of 30–35 wk-old control (*n* = 10) and mb1-Cre (*n* = 47) mice. Unpaired t-test with Welch’s correction. **c** Dot plot representation of the quantification of CD5 + IgM+ B cells (CD19+) in the blood of mb1-Cre mice (*n* = 14) and its evolution over time. Data points were adjusted to an exponential growth. **d** Kaplan-Meier survival plot of Rosa26-*RRAS2*^fl/fl^xmb1-Cre (mb1-Cre+) mice (*n* = 22) and WT control C57BL/6 J mice (*n* = 40) allowed to age in the same housing conditions. Median survival for mb1-Cre = 13.57 months; median survival for WT controls = 32.39 months. *P* < 0.0001, long-rank Mantel-Cox test and Gehan-Breslow-Wilcoxon test. **e** Experimental setup for the adoptive transfer experiment. Total B cells from mouse donors bearing the hematopoietic cell marker allele CD45.2 were purified and inoculated i.v. in the tail vein of sublethaly irradiated wild type recipient of the same strain (C57BL/6) but bearing the CD45.1 allele. Donor control mice refer to Rosa26-*RRAS2*^fl/fl^ without Cre recombinase 11 wk-old and the donor problem mice corresponded to 16 wk-old Rosa26-*RRAS2*^fl/fl^ x mb1-Cre. **f** Quantification by flow cytometry of the expression of CD45.2 and CD5 within the CD19+ population of inoculated CD45.1+ mice (*n* = 8) and bled at the indicated time points. In this figure, control mice refer to Rosa26-*RRAS2*^fl/fl^ without Cre recombinase. Two-way ANOVA test. **g** PCR results of using specific forward oligonucleotides for different V_H_ families and a constant reverse oligonucleotide against the J region of the immunoglobulin heavy chain gene. The specific V_H_ families analyzed in each case are indicated on top of each section of the agarose gel. H_2_O is used as a negative control. In each V_H_ family, BM corresponds to bone marrow cells of a wild-type 12 wk-old mouse, T corresponds to sorted naïve thymocytes (no recombination) from a wild type 6 wk-old mouse, and each column corresponds to the spleen of an individual mb1-Cre mouse of ages between 35 and 40 weeks. **h** Representative flow cytometry plot showing the presence of GFP^low^ and GFP^high^ populations in the spleen of a 30 wk-old Rosa26-*RRAS2*^fl/fl^xmb1-Cre mouse. **i** RT-qPCR for the differentially expressed *RRAS2* gene in the CD19 + CD5+ GFP^high^ and GFP^low^ populations sorted from spleens of *n* = 7 30–35 wk-old Rosa26-*RRAS2*^fl/fl^xmb1-Cre mice compared to follicular B cells of *n* = 6 30 wk-old WT mouse controls. One-way ANOVA test. **j** Dot plot representation of GFP^high^ CD5+ leukemic B cell evolution in blood from mb1-Cre mice over time, showing each mouse individually (*n* = 14). Data points were adjusted to a linear fit. **k** Paraffin-embedded lung tissue section stained with hematoxylin-eosin, showing representative lymphocytic infiltration in one out of three 35 wk-old Rosa26-*RRAS2*^fl/fl^xmb1-Cre mice. **l** Percentage of IgM + CD5+ B cells within the lymphoid population infiltrating the lungs of *n* = 2 35 wk-old Rosa26-*RRAS2*^fl/fl^xmb1-Cre mice compared to *n* = 2 32 wk-old Rosa26-*RRAS2*^fl/fl^ control ones. **m** Bar plot of the distribution of lymphoid cells between the GFP^low^ and GFP^high^ populations in the lungs and lymph nodes (LN) of 20 wk-old Rosa26-*RRAS2*^fl/fl^xmb1-Cre mice (*n* = 2)

We next set out to determine if overexpression of human *RRAS2* caused the generation of a B cell leukemia in a B cell-intrinsic manner. To this end, we crossed Rosa26-*RRAS2*^*fl/fl*^ mice with mb1-Cre mice which express Cre recombinase specifically in B cells starting at an early precursor phase [23]. Those mice accumulated a large number of CD19^+^IgM^+^CD5^+^ B cells in the blood, showing that the development of the B cell leukemia is B cell-intrinsic (Fig. 2a and b).

The presence of increasingly large numbers of IgM + CD5+ B cells in the blood is a feature of human CLL [3]. We found that the number of leukemic CD19^+^IgM^+^CD5^+^ B cells in the blood progressed with age in nearly all Rosa26-*RRAS2*^*fl/fl*^xmb1-Cre mice (Fig. 2c). The time-dependent increase in leukemic cell numbers in the blood may be responsible for the reduced life span of Rosa26-*RRAS2*^*fl/fl*^xmb1-Cre mice (t1/2 = 13 months) compared to that of wild type controls (t1/2 = 32 months, Fig. 2d). Another feature of some patients with CLL is the increased concentration of IgM in serum, something that was also detected in Rosa26-*RRAS2*^*fl/fl*^xmb1-Cre mice (Fig. S1g). A Giemsa staining of a blood smear showed the presence of enlarged lymphocytes with higher cytoplasmic content in Rosa26-*RRAS2*^*fl/fl*^xmb1-Cre mice than those of WT control mice (Fig. S1h). Along these lines, the analysis by flow cytometry of the Forward Scatter parameter showed that the CD19 + CD5+ cells in blood are larger than normal (Fig. S1i and k). However, the analysis of cells entering the cell cycle in a period of 24 h by i.v. administration of the thymidine analogue BrdU showed that only 0.2% of the CD19 + CD5+ cells in the blood had incorporated BrdU (Fig. S1j and k), indicating that, in spite of being large, the CD19 + CD5+ leukemic cells in blood are not progressing through the cell cycle and are not lymphoblastic. The low rate of proliferation in blood correlate with findings in human CLL suggesting that circulating leukemic B cells are not in proliferation [32].

To demonstrate that *RRAS2*^*fl/fl*^xmb1-Cre mice develop a CLL leukemia that can be transplanted to normal mice, we carried out adoptive transfer experiments in which sub-lethally irradiated wild-type mice expressing the hematopoietic cell allele marker CD45.1 were transferred with total B cells from young Rosa26-*RRAS2*^*fl/fl*^xmb1-Cre mice or Rosa26-*RRAS2*^*fl/fl*^ controls. Donor mice bear the CD45.2 allele and therefore transferred cells can be distinguished from endogenous ones according to the expression of the CD45.2 marker (Fig. 2e). We followed the progression of the leukemic CD19^+^IgM^+^CD5^+^ B cell population in the blood of the transferred mice and observed a progressive increase over time of the percentage of leukemic cells within the total (donor plus acceptor) B cell population (Fig. 2f). Those experiments demonstrated that the abnormal B cell population (or its precursors) can be transplanted and expands in non-diseased recipient mice, adding further support to the notion that the CD19^+^IgM^+^CD5^+^ B cell population is a CLL.

We also carried out an analysis of V_H_ family usage in 9 independent Rosa26-*RRAS2*^*fl/fl*^xmb1-Cre mice by PCR of genomic DNA. We found that, unlike total bone marrow cells from a wild-type donor (BM, Fig. 2g), which did not result in amplification of any particular band (except for V_H_ family J558), sorted CD19^+^IgM^+^CD5^+^ B cells from mice with *RRAS2* overexpression had overrepresentation of some V_H_ families resulting in a pattern of bands that was specific to each individual mouse. Those data indicate that CD19^+^IgM^+^CD5^+^ B cells are oligoclonal and, therefore, additionally support the notion that *RRAS2*-overexpressing mice develop a CLL leukemia. Interestingly, the oligoclonal BCR repertoire in the analyzed mice included families known to mediate autoimmunity, i.e., VH11 and VH12 [33, 34]. Autoantigens have been shown to select B cells with exacerbated BCR signaling, hence inducing aggressive progression of CLL mouse models [35].

The expression of markers, the oligoclonality, the transferability, the slow steady increase of non-blastic, non-mitotic, B cells in the blood are all features of human CLL. Therefore, the results presented in Figs. 1 and 2 demonstrate that overexpression of human *RRAS2* provokes the development of a CLL in mice, suggesting a cause-effect relationship for *RRAS2* overexpression in the human disease.

### Time-dependent increase of *RRAS2* expression in leukemic B cells

Taking advantage of the GFP marker included in the *RRAS2* overexpression cassette in the Rosa26 locus (Fig. S1b), we could track cells in the blood and lymphoid organs of Rosa26-*RRAS2*xSox2-Cre and Rosa26-*RRAS2*xmb1-Cre mice. Interestingly, in Rosa26-*RRAS2*xmb1-Cre mice, two distinct populations of CD19^+^ B cells with approximately a 10-fold difference in GFP expression were detected in blood: a GFP^low^ and a GFP^high^ population (Fig. 2h). Likewise, the two populations of GFP^low^ and a GFP^high^ B cells were also identified in the lymphoid organs of Rosa26-*RRAS2*xSox2-Cre mice (Fig. S2a). By contrast, T cells from those mice had a predominant GFP^low^ population. Interestingly, the presence of the GFP^high^ population was more abundant within the leukemic CD5^+^ cell pool than within the follicular CD23^+^ B cell population (Fig. S2b). In the leukemic, CD5^+^ B cells, 80% were GFP^high^. Those results suggested that the enrichment in GFP^high^ cells was taking place only within the leukemic B cell population.

To study this phenomenon, we first determined if the levels of GFP expression were correlated with those of *RRAS2* expression. We sorted each of the GFP populations from total CD19^+^CD5^+^ cells from Rosa26-*RRAS2*xmb1-Cre mouse spleens and measured *RRAS2* mRNA expression by RT-qPCR. We found that *RRAS2* was overexpressed in the GFP^low^ population by 3.8-fold in comparison with follicular B cells from wild-type mice, whereas overexpression reached a mean of 20-fold in the GFP^high^ population (Fig. 2i). A Western blot analysis of whole cell lysates of sorted GFP^low^ and GFP^high^ leukemic cells showed that R-RAS2 protein expression was also much higher in GFP^high^ than in GFP^low^ cells (Fig. S2c). Therefore, *RRAS2* in CD5+ B cells is overexpressed in two populations, one with moderate and the other with very high (20-fold) levels. Interestingly, we found that the GFP^high^ /GFP^low^ proportion within the CD19^+^CD5^+^ B cell population in the blood increases with age when mice are analyzed individually by periodic bleedings (Fig. 2j and Fig. S2d). In some mice, we detected infiltration of non-hematopoietic tissues such as kidneys, lungs and liver by lymphoid cells in old Rosa26-*RRAS2*xmb1-Cre mice. For instance, in 3 out of 17 one-year-old males, we detected perivascular infiltration of the lungs by lymphoid cells that were not forming follicles (Fig. 2k). The analysis by flow cytometry indicated that those infiltrates were constituted mainly by CD19^+^CD5^+^ leukemic B cells (Fig. 2l) with an overrepresentation of GFP^high^ cells (Fig. 2m). These data suggest that CD19^+^CD5^+^ B cells with the highest expression of *RRAS2* are the ones with the highest metastatic potential. Those data suggest that the age-dependent enrichment in CD19^+^CD5^+^ B cells with the highest expression of GFP is the result of a selective pressure that favors cells with the highest expression of *RRAS2* and metastatic potential. Furthermore, the analysis of B cell markers in GFP^high^ and GFP^low^ cells in spleen shows that GFP^high^ are the cells with the highest proportion of CD21-CD23-CD19+ B cells (53%, Fig. S2e) and downregulated IgD, CD21, and B220. Interestingly, B220 downregulation is typical of B1 CD5+ B cells which are suspected to be the origin of leukemic cells in an Eμ-TLC1 mouse model of CLL [36]. In an attempt to determine if CD19 + CD5+ leukemic B cells in Rosa26-*RRAS2*xmb1-Cre mice derive from B1 CD5+ cells, we quantified the number of B cells in the peritoneum, a site rich in B1a and B1b cells, according to the expression of the CD11b and CD5+ markers. We found that leukemic cells were CD11b + CD5+, markers of B1a cells (Fig. S3a). However, although the number of B cells with those markers was increased 7-fold compared to WT controls, the number of cells with the same markers was increased by 10-fold in spleen. Therefore, it is not possible to assign a B1a origin in the peritoneum or the spleen according to cell numbers. IgM expression was more heterogeneous in the leukemic CD11b + CD5+ population of the peritoneum than in B1a cells of control WT mice (Fig. S3b). On the other hand, B220 was low or negative in both peritoneal WT B1a cells and leukemic cells (Fig. S3c). This population was enriched in the spleen of leukemic mice compared to WT controls. B1 cells emerge early in life from precursors in the liver. We compared the number of CD19 + CD5+ cells and the expression of different markers in 2 week-old mice of Rosa26-*RRAS2*xmb1-Cre mice with age-matched controls. We found that the liver of leukemic mice was enriched in CD19 + CD5+ negative for B220, CD21, CD23 and expressing intermediate levels of CD24 and high levels of CD38 (Fig. S4). However, the same phenotype and even bigger number of cells were already present in the spleen. Therefore, we cannot conclude if leukemic CD19 + CD5+ in *RRAS2*-overexpressing mice have a B1a origin in the liver or if they develop from mature B cells in the spleen.

To further compare the GFP^low^ and GFP^high^ B cell populations, and these with leukemic CD19 + CD5+ B cells and normal follicular B cells, we carried out an RNAseq analysis of the transcriptome. We compared the following cell populations: follicular B cells sorted from spleens of six wild-type C57BL/6 mice; sorted CD19 + CD5+ leukemic B cells from spleens of six independent Rosa26-*RRAS2*xmb1-Cre mice; sorted normal B follicular B cells from the spleens of two Rosa26-*RRAS2*xmb1-Cre mice; sorted CD19 + GFP^low^ and sorted CD19 + GFP^high^ B cell populations from two Rosa26-*RRAS2*xmb1-Cre mice. Gene expression data is summarized in Table S1. Principal component analysis shows a clustering of follicular B cells from Rosa26-*RRAS2*xmb1-Cre mice together with follicular B cells from wild-type C57BL/6 mice and of GFP^low^ and GFP^high^ B cell populations with leukemic B cells (Fig. S5a). A comparison of the most differentially expressed genes in GFP^low^ versus GFP^high^ B cell populations with their expression in leukemic (CD19 + CD5+) and normal follicular B cells populations shows that GFP^high^ B cells are more closely related to leukemic cells than GFP^low^ B cells (Fig. S2f).

### Overexpression of wild-type R-RAS2, but not expression of an oncogenic mutant of R-RAS2, induces CLL

Unlike for *KRAS*, oncogenic mutations in *RRAS2* are rarely found in human cancer (Fig. 1a and b). However, the oncogenic mutation Q72L in *RRAS2* has been found as a hotspot [37]. Indeed, of the 218 cancer samples (out of 45,604) with gene alterations in *RRAS2*, the missense mutation Q72L was the most frequent (www.cbioportal.org and Fig. S6a). To determine if mice expressing the Q72L mutant of *Rras2* also developed CLL, we crossed knock-in *Rras2*(Q72L)^fl/fl^ mice bearing a repeated and inverted exon 3 in the *Rras2* locus with mb1-Cre mice to specifically exchange wild-type exon 3 for the mutant exon in B cells. The transcription of the *Rras2* locus bearing the Q72L mutation was equivalent to that of B cells from WT control mice and much lower than that of B cells from Rosa26-*RRAS2*xmb1-Cre mice (Fig. S6b). We analyzed 14-month-old mice and their control littermates (negative for mb1-Cre) for evidences of a CLL and found that the number of B cells in spleen and blood (Fig. S6c), the presence of CD19 + CD5+ cells in blood (Fig. S6d), and the distribution of B cells among follicular, marginal zone and CD21^−^CD23^−^ B cells (Fig. S6e) were within normal limits. These data suggested that overexpression of wild-type *RRAS2* and not an oncogenic mutation drives the development of CLL in mice.

### A highly conserved pattern of somatic mutations accompanies the development of *RRAS2*-driven CLL

The comparison of the transcriptome of six wild-type follicular B cell samples and seven independent leukemic samples using the Ingenuity Pathway Analysis (IPA) software revealed a strong signature of pathway alterations related to molecular mechanisms of cancer (Fig. S5b), thus confirming the malignant nature of the CD19 + CD5+ B cells expanding in Rosa26-*RRAS2*xmb1-Cre mice. In addition, there is a signature of increased mTOR activity which could be expected given the capacity of R-RAS2 to activate this pathway in B cells (Fig. S7, and [14].

**Fig. 3:**
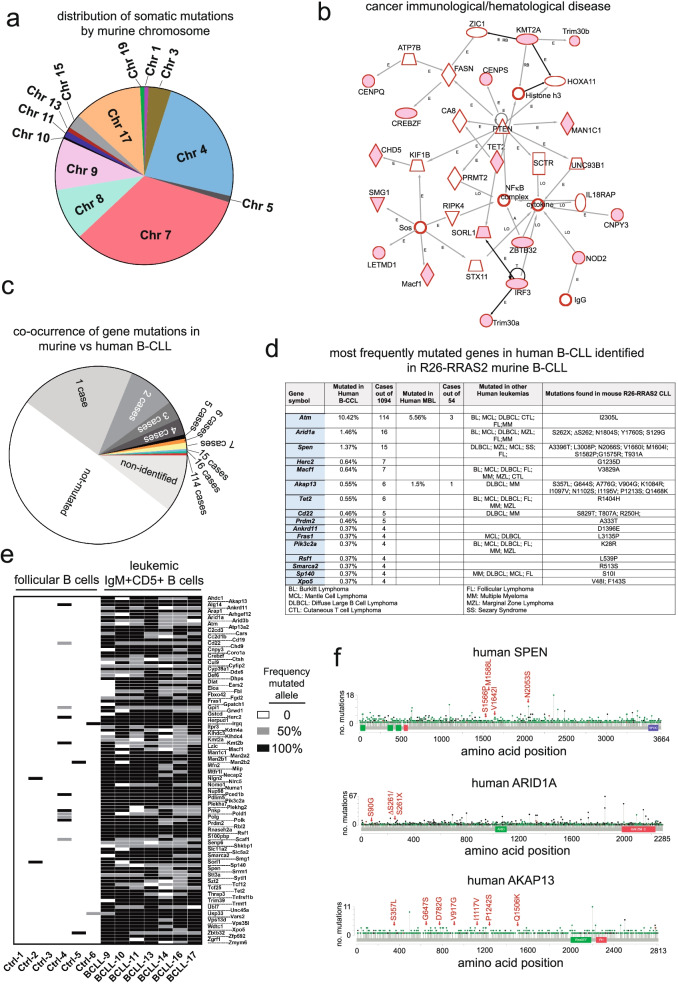
*RRAS2*-driven CLL harbors a highly conserved pattern of somatic mutations. **a** Pie chart representation of the somatic mutation distribution from CD19 + CD5+ leukemic cells by chromosome. Spleens from seven Rosa26-*RRAS2*^fl/fl^xmb1-Cre mice were included in this analysis (ages in panel **e**). **b** Ingenuity Pathway Analysis (IPA) diagram of the immunological and hematological neoplastic signature generated from the 270 mutations present in all seven different Rosa26-*RRAS2*^fl/fl^xmb1-Cre mice. **c** Pie chart representation of the co-occurrence of gene mutations in murine CLL vs human CLL. **d** Summary of most frequently mutated genes in 1094 cases of human CLL also identified in Rosa26-RRAS2 murine CLL. The panel also shows each gene’s condition in MBL (monoclonal B lymphocytosis) and other human leukemias (BL:Burkitt Lymphoma; MCL: Mantle Cell Lymphoma; DLBCL: Diffuse Large B Cell Lymphoma; CTL:Cutaneous T cell lymphoma; FL: Follicular Lymphoma; MM: Multiple Myeloma; MZL; Marginal Zone Lymphoma; SS: Sezary Syndrome). **e** Heatmap of mutation rates of 107 mutated genes detected in CD19 + CD5+ leukemic cells from seven Rosa26-*RRAS2*^fl/fl^xmb1-Cre independent mice (age of mice: Ctrl 1–3, 12 wk.; Ctrl 4–6 25 wk.; BCLL-9, 104 wk.; BCLL10–11 and BCLL16–17, 54 wk.; BCLL13–14, 32 wk). Black: homozygous; grey: heterozygous; white: unmutated. **f** Schematic representation of registered missense mutations in *SPEN*, *ARID1A* and *AKAP13* in human CLL patients (green dots: missense mutation; black dots: truncating mutations; orange dots: splicing site mutation). Red text indicates mutations found in Rosa26-*RRAS2*^fl/fl^xmb1-Cre mice which are also present in human genes

Nonetheless, the most striking finding resulting from the analysis of RNAseq data was that CD19 + CD5+ leukemic cells from the spleens of seven independent mice showed the consistent presence of somatic mutations in 270 genes in all mice. Those mutations corresponded mostly to missense mutations and less so to frameshifts or nonsense mutations (Table S2). The mutated genes concentrated on chromosomes 7 and 4, followed by chromosomes 8, 9 and 17 (Fig. 3a). Chromosomes 2, 6, 12, 14, 16, 18 and both sex chromosomes contained no mutations in mRNA-encoding genes. IPA analysis of the 270 mutated genes identified a signature of genes related to immunological and hematological neoplasia (Fig. 3b) and to immunological development (Fig. S7); including genes such as *Atm*, *Kmt2a*, *Macf1*, *Tet2*, *Akap13*, *Cd19*, *Cd22* and *Polk*. Finally, mutations in genes associated with retinoblastoma, cyclins and *Atm*, suggest that leukemic cells have unlocked the G1-S checkpoint (Fig. S7).

Out of the 270 genes found mutated in our RNAseq analysis of murine CLL cells, a total of 107 were found mutated within a cohort of 1094 human CLL patients and 8 within a cohort of 54 human MBL patients (www.cbioportal.org). Of those, the most frequently mutated gene in human CLL that is also found in our murine CLL model is *ATM*, with 114 cases out of 1094 patients (Fig. 3c). A table with the list of genes identified in four or more human CLL patients shows that, in addition to *ATM*, other known tumors suppressors such as *ARID1A*, *AKAP13*, *PRDM2*, *SPEN*, *SMARCA2* and *TET2* are also mutated (Fig. 3D). Within this set of genes, there are the epigenetic regulators *ARID1A* and *SMARCA2*, which form part of the chromatin remodeling complex, SWI/SNF, and the DNA damage repair genes *ATM* and *HERC2*. Most of the genes mutated in Rosa26-*RRAS2* CLL leukemias and human CLL leukemias are also found mutated in other hematological cancers of B and T cells (Fig. 3d). A striking observation of our RNAseq analysis is that the vast majority of the 107 genes mutated in R26-RRAS2 CLL, that are also found mutated in human CLL, were mutated in homozygosity (100% of the sequences) or heterozygosity (50% of the sequences) in leukemic cells from each of the seven independent mice (Fig. 3e). Those data suggest a strong selective pressure for a combination of accompanying gene mutations that drive CLL development in mice that overexpress wild-type *RRAS2*, thus reinforcing the idea previously established that B cells with the highest *RRAS2* expression (GFP^high^, Fig. 2 and Fig. S2) are selected during evolution of the leukemia. Another piece of evidence supporting the idea of selection is that many genes contain multiple missense mutations in their mRNAs in 50% or even 100% of the sequences, suggesting an evolution of B cells with a progressive number of mutations in the same genes. An example is shown for the genes *Spen*, *Arid1a* and *Akap13* that have multiple mutations in their coding sequences (Fig. 3f and Table S2). Those positions are also found mutated in human CLL.

### R-RAS2 is complexed with the BCR in leukemic cells and is required for proliferation and formation of tumors in xenografts by human CLL

**Fig. 4:**
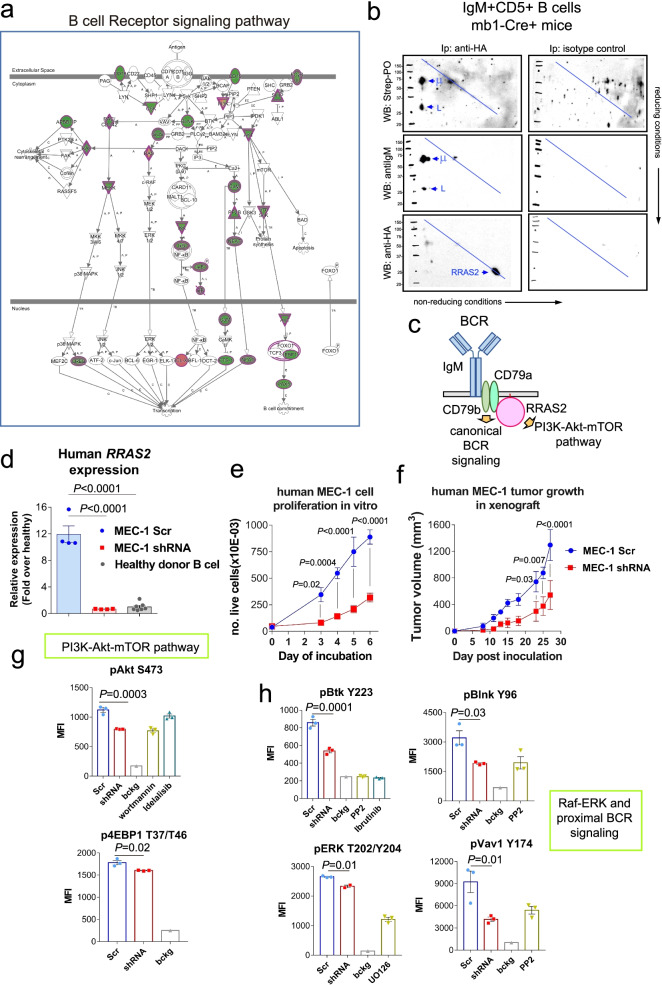
R-RAS2 is associated with the BCR in leukemic cells and is required for human CLL cell proliferation. **a** Ingenuity Pathway Analysis (IPA) of differentially expressed genes in leukemic versus normal follicular B cells. Pink-filled symbols: upregulated genes. Green-filled: downregulated genes. Double circle: protein complex; horizontal ellipse: transcription regulator; vertical ellipse: transmembrane receptor, diamond: enzyme; trapezium: transporter; triangle: phosphatase; inverted triangle: kinase; circle: other. Relationship labels: A: activation; B: binding; C: causation; CO: correlation; E: expression; EC: enzyme catalysis; I: inhibition; L: molecular cleavage; LO: localization; M: biochemical modification; miT: microRNA Targeting; P: phosphorylation/dephosphorylation; PD: protein-DNA binding; PP: protein-protein binding; PR: protein-RNA binding, RB: regulation of binding; RE: reaction; T: transcription; TR: translocation; UB: ubiquitination. **b** Western Blot of two-dimensional (2D) gel electrophoresis under non-reducing/reducing conditions of purified CD19 + CD5+ cells from the spleen of a 45 wk-old Rosa26-*RRAS2*^fl/fl^xmb1-Cre mouse. Left: co-immunoprecipitation of R-RAS2-interacting components using an anti-HA antibody. Right: isotype IgG2b control. Membranes were serially incubated with streptavidin-PO, anti-IgM and anti-HA. The positions of molecular weight markers are indicated to the left of each membrane. The blue line represents the mobility of proteins without inter-chain disulfide bridges. **c** Schematic representation of R-RAS2 interaction with the B-cell receptor and its downstream effects on canonical BCR signaling and the PI3K-Akt-mTOR pathway. **d** RT-qPCR analysis of *RRAS2* expression in MEC-1 cell line transduced with scrambled control lentiviral particles (blue), shRNA for human *RRAS2* (red), and expression of *RRAS2* in healthy peripheral blood lymphocytes (grey). One-way ANOVA test. **e** In vitro proliferation assay of *RRAS2* knockdown MEC-1 cells (red) and control (blue). Data show means ± SEM from three biological replicates. Two-way ANOVA test, row factor. **f** Tumor growth in immunodeficient mice. 10 × 10^6^ transduced MEC-1 cells per animal were subcutaneously injected. Data show means ± SEM from a total of eight mice. Two-way ANOVA test, row factor. **g** & **h** Flow cytometry analysis of Raf-ERK pathway and proximal BCR signaling components ERK (T202/Y204), VAV (Y174), BTK (Y223) and BLNK (Y96) phosphorylation in MEC-1 cell line transduced with scrambled control lentiviral particles (blue), shRNA for human *RRAS2* (red) and treated with Src-kinases inhibitor PP2 20 μM, Btk inhibitor Ibrutinib 10 μM and MEK inhibitor U0126 10 μM

Human CLL is characterized by the presence of recurrent “stereotyped” BCRs often with similar or identical sequences in the IgHV chain [6, 38]. This indicates that BCR signaling is fundamental for the development of CLL with the existence of stereotyped BCRs, suggesting the existence of common antigens (probably autoantigens) as initial triggers of the expansion of leukemic clones. Indeed, expression of the CD5 marker, characteristic of CLL, is induced by BCR signaling [39]. Our IgHV usage data (Fig. 2g) shows oligoclonality in the BCR repertoire of CLL cells emerging in Rosa26-*RRAS2*xmb1-Cre mice. In addition, IPA analysis of gene transcription in leukemic versus normal follicular B cells (Table S1) shows a strong signature of active BCR signaling (Fig. 4a), suggesting that the BCR is actively signaling in leukemic cells with activation of the PI3K-Akt-mTOR, the NFκB and the NFAT pathways, among others.

We previously demonstrated that R-RAS2 is constitutively associated to both the BCR and the TCR of normal B and T cells, respectively [12], and that it is an activator of PI3K and mTOR pathways [12, 14]. Therefore, we investigated if R-RAS2 is also physically associated to the BCR in leukemic cells. To this end, we biotin-labelled all plasma membrane proteins of purified CD19 + CD5+ leukemic cells from spleens of Rosa26-*RRAS2*xmb1-Cre mice using a membrane-impermeable biotinylation reagent and immunopurified R-RAS2 protein using an anti-HA antibody. The immunoprecipitates were subjected to two-dimensional SDS-PAGE under non-reducing/reducing conditions and immunoblotting was carried out with streptavidin-peroxidase for identification of total membrane proteins co-purifying with R-RAS2. Two biotinylated proteins corresponding in size to the IgH μ chain and the IgL chain were detected (Fig. 4b). Those two proteins were specifically detected with an anti-IgM (H + L) reactive antibody, thus demonstrating that R-RAS2 is constitutively interacting with the BCR in leukemic cells. By interacting with the BCR, R-RAS2 could be placed just downstream of this receptor in the activation of the PI3K-Akt-mTOR pathway and other canonical BCR signaling pathways (Fig. 4c), as suggested by the RNAseq data (Fig. 4a). Of note, we previously placed R-RAS2 as a direct BCR effector important for the transmission of tonic BCR signals required for survival and homeostatic cell proliferation in the absence of antigen [12]. This property of R-RAS2 aligns with a proposed antigen-independent signaling role for the BCR in CLL [8].

In order to highlight the causal relationship between *RRAS2* overexpression and appearance of CLL, we next used the human CLL cell line MEC-1 to determine if R-RAS2 overexpression is required for the activation of BCR signaling pathways and for proliferation and survival of human CLL cells. MEC-1 cells overexpress *RRAS2* mRNA at values 12-fold higher than those of B cells from peripheral blood of healthy human donors (Fig. 4d). Transducing MEC-1 cells with a lentiviral construct expressing a shRNA for human *RRAS2* reduced the expression of this gene to levels close to those of normal B cells (Fig. 4d). The *RRAS2* knockdown MEC-1 cells proliferated more slowly than their control counterparts in vitro (Fig. 4e) and, more importantly, produced smaller tumors than control MEC-1 cells when transplanted subcutaneously into lymphopenic mice (Fig. 4f). These results suggest that high R-RAS2 expression is required for human CLL cell proliferation in vitro and in vivo. Furthermore, the analysis by phosflow cytometry of knockdown and control cells showed that high R-RAS2 expression in MEC-1 cells is required to activate the PI3K-Akt-mTOR (pAkt and p4EBP1) pathway (Fig. 4g), as well as the proximal BCR signaling (pBlnk, pVav1 and pBtk) and other canonical pathways (MAPK/ERK) (Fig. 4h). Therefore, those data suggest that *R-RAS2* expression is mediating BCR signaling in a human CLL cell line, as well as proliferation in vitro and in vivo.

To determine if R-RAS2 is also mediating BCR signaling in leukemic cells from Rosa26-*RRAS2*xmb1-Cre mice, we first analyzed those cells by phosflow cytometry in parallel to B cells from WT controls and to B cells from *Rras2*(Q72L)^fl/fl^ xmb1-Cre mice, which do not develop CLL (Fig. S6a-f). We found that leukemic B cells overexpressing R-RAS2 present significantly higher activation of the PI3K-Akt-mTOR pathway (pAkt, pS6 and pEBP1) and also higher activation of proximal BCR-signaling (pBlnk) and Raf-ERK pathway (pERK) than normal control follicular B cells (Fig. S6f). Activation of the PI3K-Akt-mTOR, Raf-ERK and BCR signaling pathways was also higher in leukemic B cells than in B1a, B1b and marginal zone (MZ) normal B cells (Fig. S6g), being MZ the B cell population with the highest constitutive activation of those pathways. In addition, the results of Fig. S6 suggest that *Rras2*(Q72L)^fl/fl^ xmb1-Cre mice expressing a constitutively active “oncogenic” mutant of R-Rras2 do not develop CLL because they do not activate those BCR signaling pathways. *Rras2*(Q72L)^fl/fl^ xmb1-Cre mice do not develop any detectable malignancy, whereas mice that express the *Rras2*(Q72L) mutant in all tissues (tamoxifen-regulated Cre-ERT2-regulated mouse strain [iCre-*Rras2*Q72L]) do develop a form of T-cell acute lymphoblastic leukemia (T-ALL) in 100% of mice, but not CLL (Fernandez-Pisonero et al., under revision).

### Wild-type *RRAS2* is overexpressed in human CLL and correlates with parameters of poorer prognosis

**Fig. 5:**
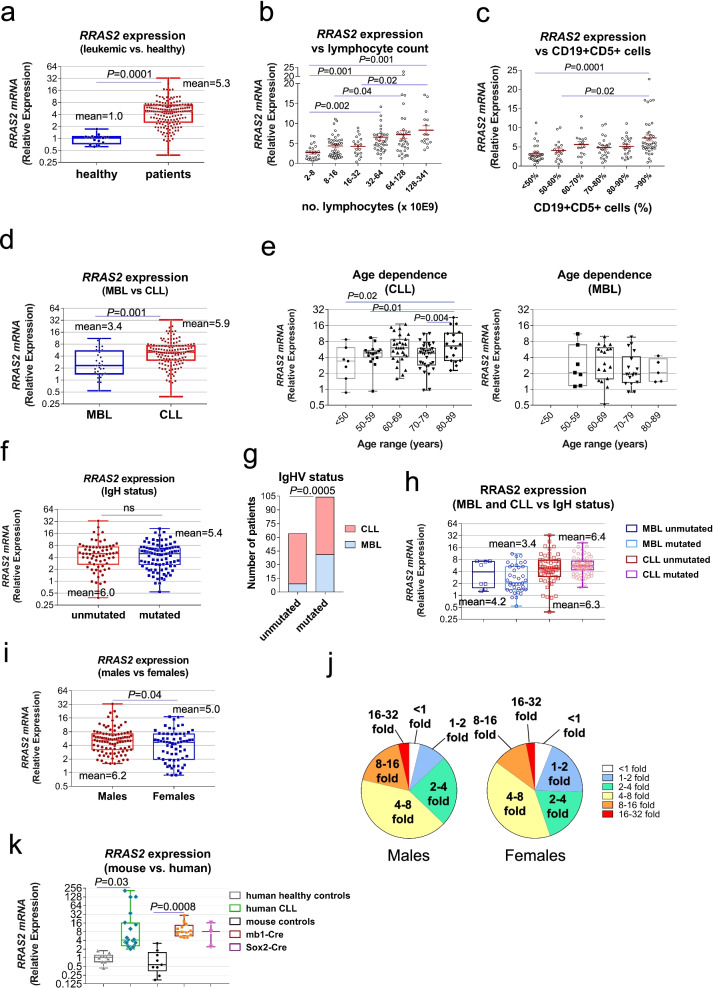
*RRAS2* expression is increased in CLL patients and associated to more aggressive disease. **a** Box and whiskers plots showing all points and median value of RT-qPCR data of *RRAS2* mRNA expression in PBMCs from healthy individuals and from MBL and CLL patients. Expression data is normalized to the mean value of healthy blood donor values (*n* = 17). Two-tailed unpaired t-test with Welch’s correction. **b** RT-qPCR analysis of *RRAS2* mRNA expression compared to the lymphocyte count in the blood of CLL patients. Patients are classified according the number of total lymphocytes in blood (in ranges, in the x-axis). Values represent the mean ± SEM. All datapoints are represented. One-way ANOVA test. **c** RT-qPCR analysis of *RRAS2* expression compared to the percentage of CD19 + CD5+ cells (in ranges in the x-axis) in the blood of CLL patients. All datapoints are represented. One-way ANOVA test. **d** Box and whiskers plots showing all points and median value of RT-qPCR data of *RRAS2* mRNA expression in blood from MBL and CLL patients. Expression data is normalized to the mean value of healthy blood donor values. Two-tailed unpaired t-test with Welch’s correction. **e** Box and whiskers plots showing all points and median values of RT-qPCR data of *RRAS2* mRNA expression versus age of the patient at diagnosis (in 10-year intervals) and divided according to the diagnosis as CLL or MBL. One-way ANOVA test. **f** Box and whiskers plot showing all points and median value of RT-qPCR data of *RRAS2* mRNA expression in CLL patients classified according to the expression of a mutated or an unmutated IgH gene. ns, not significant (two-tailed unpaired t-test with Welch’s correction). **g** Contigency test of the distribution of mutated and unmutated IgHV gene within MBL and CLL patients in our study cohort. **h** Box and whiskers plot showing all points and median value of RT-qPCR data of *RRAS2* mRNA expression in our study cohort classified according to MBL vs CLL diagnosis and having mutated or unmutated IgHV. **i,** Box and whiskers plots showing all points and median value of RT-qPCR data of *RRAS2* mRNA expression in blood from all patients in our study cohort classified according to male or female sex. Two-tailed unpaired t-test with Welch’s correction. **j** Pie chart diagram of the relative fold variations in the expression of *RRAS2* mRNA in male and female CLL patients, classified in fold intervals. 1 is the mean of expression in healthy blood donors. **k** Box and whiskers plot showing all points and median value of RT-qPCR *RRAS2* mRNA expression in blood from healthy individuals and CLL patients, and in spleen B cells from control mice, mb1-Cre mice, and Sox2-Cre mice. One-way ANOVA test). In all panels, when there is no distinction between MBL and CLL patients, CLL refers to both groups

Considering that Rosa26-*RRAS2*xmb1-Cre and Rosa26-*RRAS2*xSox2-Cre mice develop CLL, that the MEC-1 human CLL cell line overexpresses *RRAS2* and that these cells require *RRAS2* expression for proliferation and tumor formation, we set out to assess *RRAS2* mRNA levels in our own cohort of previously untreated CLL patients to reinforce the metadata (Fig. 1c and S1a) and further analyze the relationship between *RRAS2* overexpression and human CLL. In the cohort (*n* = 178), the average age was 69 years and 60% were males (Table S3). A 63% were in early stages of the disease, i.e., Binet stage A or Rai stage 0, and a total of 52 were classified as having a premalignant mononuclear B cell lymphocytosis (MBL) condition, whereas 38% were IGHV-UM CLL with worse prognosis [40]. Importantly, in line with repository data and correlating with our *RRAS2*-overexpressing mice, we found a significant increase in *RRAS2* mRNA levels in CLL patients compared to healthy subjects, with a mean of 5.3-fold higher expression in CLL patients (Fig. 5a). A classification of patients according to the number of total lymphocytes in blood (Fig. 5b) and the percentage of malignant CD19 + CD5+ B cells (Fig. 5c) showed a direct correlation with overexpression of *RRAS2*, i.e., the group of patients with highest lymphocytosis and malignant cell content has the highest overexpression of *RRAS2*. The classification of patients that present premalignant MBL versus full-blown CLL is based on the degree of lymphocytosis (threshold at 5 × 10^6^ lymphocytes/mm^3^). Both conditions occurred upon overexpression of *RRAS2*, though overexpression in CLL was significantly higher (5.9 fold) than in MBL (3.4 fold, Fig. 5d). Advanced age is another condition of poorer prognosis [40]. We found increased median and mean overexpression of *RRAS2* with age in full-blown CLL, but not in MBL (Fig. 5e).

The analysis of *RRAS2* expression according to the unmutated vs mutated status of the IgHV region showed a higher mean for the IGHV-UM than for the IGHV-M CLL, although those differences did not reach statistical significance (Fig. 5f). In the subject cohort, most of the samples with unmutated IgHV classified as CLL, not MBL (Fig. 5g). The grouping of leukemias according to MBL, CLL, IGHV-UM and IGHV-M showed that *RRAS2* expression correlated with CLL, regardless of IgHV status (Fig. 5h). Another risk factor in CLL is sex, for male patients carry a worse prognosis than female ones [40]. We found that leukemias from male patients had significantly higher expression of *RRAS2* than those from female patients (Fig. 5i). A classification of leukemias by fold expression intervals showed that 82% of all samples overexpressed two-fold or more *RRAS2* mRNA than B cells from healthy controls, although the distribution by sex was unequal: 13.6% of leukemias from male patients expressed lower than 2-fold levels compared to 25.4% for female patients (Fig. 5j). Conversely, 65.7% of leukemias from male patients overexpressed 4-fold or more *RRAS2* mRNA compared with 55.2% for female patients. Altogether, these data show that *RRAS2* mRNA is overexpressed in the large majority of CLL and that higher expression is associated to factors of worse prognosis such as CLL versus MBL, higher lymphocytosis, advanced age and male sex. The results of human *RRAS2* mRNA expression in our cohort of samples (Fig. 5), in repository data (Fig. 1c and S1a), and the effect of *RRAS2* overexpression in mouse B cells driving the development of CLL strongly suggest that overexpression of unmutated *RRAS2* also drives the development of human CLL. A comparative RT-qPCR expression analysis of *RRAS2* expression in human CLL and in the two mouse models studied here shows that overexpression attained in the mouse systems is within the range of *RRAS2* expression levels detected in human leukemias (Fig. 5k), thus reinforcing the idea that the driver role in human CLL is possible.

### A single-nucleotide polymorphism (SNP) in the 3′ UTR region of *RRAS2* mRNA genetically links overexpression of unmutated *RRAS2* with human CLL and more aggressive disease

**Fig. 6:**
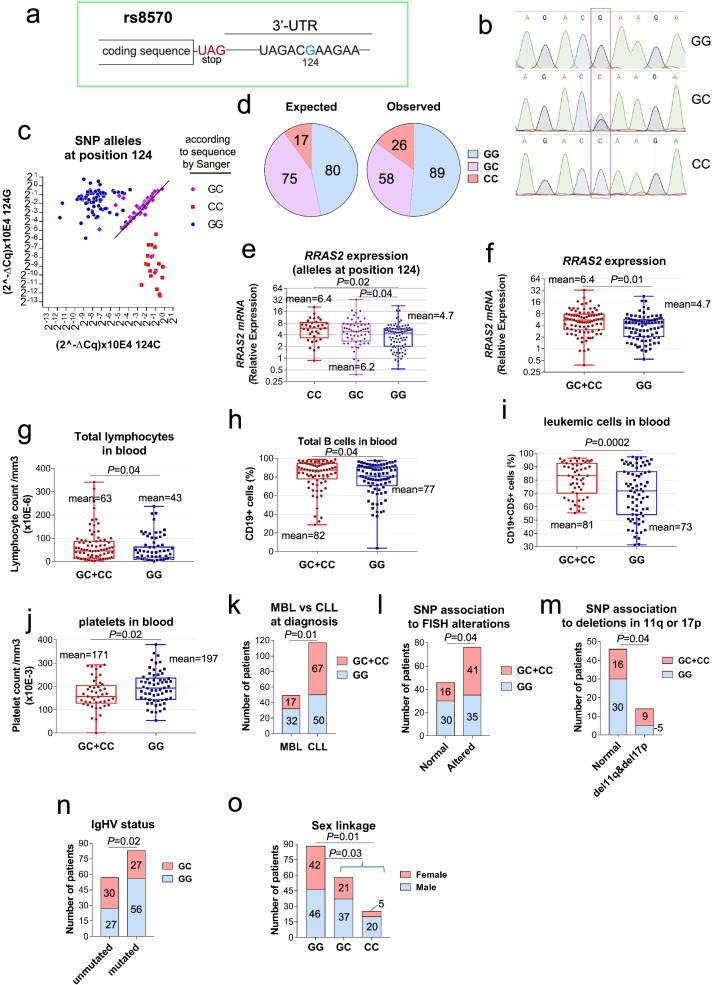
The C allele at SNP rs8570 is associated with several parameters of more aggressive disease in CLL patients. **a** Schematic representation of the rs8570 location in the 3’UTR of *RRAS2* mRNA. **b** Representative examples of the Sanger sequencing chromatograms showing the 3 possibilities of allele dosage at the position of rs8570. The rs8570 position is highlighted by a red rectangle. **c** Classification of patient samples according to the genotype at SNP rs8570 detected by a dedicated RT-qPCR strategy using specific primers for each of the 2 alleles at that position. The patients sequenced as GG, GC or CC with Sanger sequencing are plotted with dark blue, purple and orange dots, respectively. **d** Pie chart representation of the Observed versus the Expected distribution of GG, GC and CC genotypes at position of the SNP rs8570 in our study cohort of CLL patients. **e** Box and whiskers plots showing all points and median value of RT-qPCR *RRAS2* mRNA expression in blood from all patients in our study cohort classified according to GG, GC or CC genotype at position of the SNP rs8570. Ttwo-tailed unpaired t-test with Welch’s correction. **f** Box and whiskers plots showing all points and median value of RT-qPCR *RRAS2* mRNA expression in blood from all patients in our study cohort harboring none (GG) or at least one (GC and CC) 124C at position of the SNP rs8570. Two-tailed unpaired t-test with Welch’s correction. **g** Box and whiskers plots showing all points and median value of total lymphocyte count in the blood of patients harboring two G alleles or at least one C. Two-tailed unpaired t-test with Welch’s correction. **h** Box and whiskers plots showing all points and median value of the percentage of CD19+ B cells in the blood of patients harboring two G alleles or at least one C. Two-tailed unpaired t-test with Welch’s correction. **i** Box and whiskers plots showing all points and median value of the percentage of CD19 + CD5+ leukemic B cells in the blood of patients harboring two G alleles or at least one C. Two-tailed unpaired t-test with Welch’s correction. **j** Box and whiskers plots showing all points and median value of the platelet count in the blood of patients harboring two G alleles or at least one C. Two-tailed unpaired t-test with Welch’s correction. **k** Contigency test of the distribution of patients diagnosed as having MBL or CLL according to a GG or GC + CC genotype. **l** Contigency test of the distribution of all samples in our study cohort according to presenting or not chromosomal alterations by FISH and a GG or GC + CC genotype. **m** Contigency test of the distribution of samples in our study cohort according to presenting normal caryotype or presenting deletions in chromosome 11q or in chromosome 17p (or both) and a GG or GC + CC genotype. **n** Contingency test of the distribution of all samples in our study cohort according to presenting or not mutated IgHV and a GG or GC genotype. **o** Contingency test of the distribution of all samples in our study cohort according to male or female sex and a GG or GC + CC genotype

Although mutations in the coding sequence of *RRAS2* have rarely been found in human cancer, we decided to sequence the *RRAS2* mRNA in our cohort of 178 patients. We did not find any missense mutation in the coding sequence. However, in some patients we found a C nucleotide in position 124 after the stop codon in the 3’UTR, whereas the canonical sequence contains a G in that position (Fig. 6a). The C nucleotide at position 124 of the 3’UTR was the previously cataloged SNP rs8570 (from now on, 124C, in this paper). This SNP was identified within a group of SNPs of 26 genes in the MAP kinase pathway to be associated with risk of cutaneous melanoma [41]. The 124C SNP was first detected in homozygosity or heterozygosity by Sanger DNA sequencing (Fig. 6b) but later was reassessed by a RT-qPCR method using primers with mismatching positions (Fig. 6c, Methods). Using this double procedure, we calculated the frequency of GG homozygotes at position 124 of the 3’UTR in 51% of the CLL samples, 33% for GC heterozygotes and 15% for CC homozygotes. Thus, the frequency of the non-canonical 124C allele in the CLL cohort is of 32%, slightly above the 22% frequency measured in the general European population (ALFA project; https://www.ncbi.nlm.nih.gov/snp/rs8570#frequency_tab). Within our cohort of CLL patients, and according to the frequencies of GG, GC and CC genotypes at position 124, we observed a higher-than-expected frequency of CC homozygotes and lower frequency for the GC heterozygotes (Fig. 6d). The difference between Observed and Expected values indicates that the distribution of alleles is not in a Hardy-Weinberg equilibrium [42] (χ^2^ experimental = 9.62 > χ^2^ theoretical = 3.84; *p* < 0.005). The CC genotype correlated with the highest expression of *RRAS2* mRNA, whereas expression in leukemias of the GG phenotype was significantly lower than the ones of the CC genotype (Fig. 6e). Since the size of the CC group is small and mean *RRAS2* mRNA expression was very similar between the GC and CC groups, we decided to combine both groups for further analysis. The combined CC + GC group expressed significantly higher levels of *RRAS2* mRNA than the GG one (Fig. 6f). This correlated with indicators in CLL patients’ blood of more aggressive disease, such as total number of lymphocytes (Fig. 6g), higher percentage of B cells (Fig. 6h), higher percentage of leukemic CD19 + CD5+ cells (Fig. 6i) and lower number of platelets (Fig. 6j). The combined CC + GC group was significantly more represented in patients with full-blown CLL at diagnosis than in those with MBL (Fig. 6k). Several genetic alterations detected by FISH are prevalent among CLL patients, reflecting varying degrees of association with disease prognosis [43]. The presence of one or two C alleles was more common within the group of patients with chromosomal alterations determined by FISH than those with no alterations (Fig. 6l). Taking together the frequencies of deletions found in chromosome arms 11q and 17p, there is a significant association of those alterations with the presence of one or two 124C alleles (Fig. 6m). Chromosome arm 11q encodes for *ATM* and chromosome arm 17p encodes for *TP53*. Loss of any of those tumor suppressor genes is associated with poorer prognosis. We also searched for associations between the expression of the 124C allele and CLL bearing mutated or unmutated IgHV. We did not find any association between the frequency of IGHV-UM in the GG and CC groups of the cohort. However, the presence of one 124C allele (GC heterozygotes) compared to two 124G alleles (GG homozygotes) is also significantly associated to IGHV-UM (Fig. 6n). Finally, we found a clear association between male sex and the frequency of GC and CC (Fig. 6o). Expression of an unmutated IgHV and male sex are two additional factors of poorer prognosis [40]. Therefore, the SNP at position 124 of the 3′ UTR of *RRAS2* mRNA emerges as a novel prognostic factor of CLL progression and genetically proves a cause-effect relationship between *RRAS2* overexpression and human CLL.

## Discussion

Oncogenic mutations in *KRAS* or *NRAS* have been found, though in reduced numbers of CLL patients (32 out of 1308, https://www.cbioportal.org/), and no mutations have been so far identified by GWAS in *HRAS* or *RRAS2*. Therefore, 97.5% of human CLL appear without oncogenic mutations in known RAS drivers. We suggest here that overexpression of wild-type *RRAS2,* and not activating mutations in RAS members, is what drives transformation of mature B cells towards CLL, at least in 82% of these leukemias. Against the current view that RAS family members cause cancer after acquiring activating mutations, we show here that *RRAS2* does not require activating “oncogenic” mutations in its coding sequence to induce cancer. Instead, we show in a mouse model that overexpression of the wild-type form of human *RRAS2* drives the development of a CLL in 100% of mice. This is a cause-effect relationship that demonstrates that overexpression of wild type *RRAS2* is sufficient for the development of CLL in mice and suggest that 82% of the human CLL are caused by *RRAS2* overexpression. In this regard, we have found significant correlations between the highest expression of *RRAS2* in patients and relevant indicators of more aggressive disease such as number of lymphocytes in blood, percentage of leukemic cells, age dependence, male sex and CLL versus MBL. Those correlations support the notion that overexpression of *RRAS2* is important for the course of the disease in humans. The relevance of *RRAS2* overexpression has been proven here by generating an *RRAS2* knockdown variant of the CLL human cell line MEC-1, which shows impaired capacity to proliferate in vitro and to form tumors in vivo. Nonetheless, and further reflecting a driver role for wild-type RRAS2 overexpression in human CLL, SNP rs8570 in the 3’UTR of *RRAS2* is associated with poorer prognosis and higher *RRAS2* expression. At present, we have not elucidated the mechanisms that lead CLL patients that are heterozygous or homozygous for the rs8570 SNP to express more mRNA for *RRAS2.* However, as this SNP appears at the 3’UTR of the *RRAS2* locus, we could hypothesize that higher mRNA in patients bearing the SNP rs8570 is due to resistance to a miRNA. In this regard, it has been described that miR-23b inhibits metastasis of colon carcinoma cells by downregulating *RRAS2,* among other genes [44], and miR-4448 was identified in a screening of miRNA expression in B cell malignancies versus normal B cells [45]. miR-23b binds the 3’UTR of *RRAS2* near the rs8570 SNP site, whereas miR-4448 is predicted to interact with the 3′-UTR sequence at the SNP site with a score of 68 (http://www.mirbase.org). However, the participation of those or other miRNAs in differential regulation of *RRAS2* mRNA expression warrants further investigation.

Interestingly, we show here that overexpression, and not oncogenic mutation (the Q72L mutant) of *RRAS2* drives the formation of CLL in mice. This correlates with the absence of *RRAS2* missense mutations identified in human CLL both in databases and in our own cohort of 178 patients, as well as with the overexpression of at least 2-fold the normal levels of *RRAS2* mRNA in 82% of the human samples (mean and median of approximately 5-fold over healthy controls). Our previous data with *Rras2*-null mice indicate that *Rras2* is haploinsufficient, since mice heterozygous for the null allele (*Rras2*^+/−^) had the phenotype of the full knockout (*Rras2*^−/−^) and not of the wild type (*Rras2*^+/+^) [12]. In fact, heterozygous mice expressed approximately half the amount of protein in their lymphoid cells than wild-type mice. *Rras2*^−/−^ and *Rras2*^+/−^ mice have reduced numbers of follicular and marginal zone B cells due to deficient survival and homeostatic proliferation. The haploinsufficiency of *Rras2* and the B cell phenotype suggest that the total amount of R-RAS2 is tightly regulated and therefore, overexpression of *RRAS2* by two-fold, or more, in human CLL could be relevant for the human disease.

To our understanding, this is the first report demonstrating a driver role for a RAS-family member in its wild type form. Numerous studies have indicated a tumor suppressor effect of the wild type allele when in the presence of a mutant RAS allele frequently seen as a loss of heterozygosity (LOH, reviewed in [46], whereas some have reported an enhancing protumorigenic effect of the wild type allele but always in the presence of the mutated, oncogenic, one. Other studies have also shown that overexpression of NRAS promotes the development of lymphomas that are however induced by the use of chemical agents, not just overexpression of the RAS gene [47]. A more recent report has shown that increased expression of wild type *KRAS* in hematopoietic cell precursors leads to expansion of B cells but not to leukemia probably due to an exhaustion effect [48]. We explain this capacity of wild-type *RRAS2* to drive the formation of leukemias by its high guanosine nucleotide intrinsic exchange rate, higher than that of classic RAS proteins [49]. This high exchange rate in the absence of GEF regulators means that wild-type R-RAS2 protein is in the active GTP-bound state in the absence of stimuli, i.e., in basal conditions. The elevated intrinsic GDP/GTP exchange rate would be responsible for the high transformation capacity of wild-type *RRAS2* in the NIH-3 T3 fibroblast focus-formation assay [49] and for the need to keep R-RAS2 protein expression tightly regulated [12].

Perhaps one of the most interesting findings in the murine Rosa26-*RRAS2*xmb1-Cre model of CLL is the strong convergent evolution of accompanying mutations in leukemic cells from different animals in 50% or 100% of the mRNA sequences. This evolution is manifested by mutations acquired in tumor suppressor genes that have also been recurrently identified in human CLL and other B cell leukemias and lymphomas. Those genes include some involved in DNA repair (*ATM*, *ARID1A*, *SMARCA2*, *HERC2*) chromatin remodeling (*ARID1A*, *ANKRD11*, *SMARCA2*), and transcriptional repressors (*SPEN*, *TET2*, *SP140*). By contrast, a pathway that is clearly upregulated in the murine RRAS2-overexpression models of CLL is the PI3K-mTOR pathway, which is detected by both the effect on gene transcription and by the analysis of phosphorylation of key members of the pathway. This upregulation of the pathway was somewhat expected, given the ability of R-RAS2 to recruit the catalytic subunit of PI3Kδ and activate the PI3K pathway in normal B cells [50, 51].

We also show here that R-RAS2 protein is directly interacting with the BCR in murine CLL and that BCR signaling is enhanced, as determined by phosflow analysis of BCR-proximal effector proteins and by the effect on gene transcription. Interestingly, one of the genes found mutated in all analyzed samples of murine CLL, and also found mutated in human CLL, is CD22, which is a negative regulator of BCR signaling [52]. Thus, our data reinforce the notion that activated or deregulated BCR signaling is behind the development of CLL as seems also manifested by the overabundance of VH families that have been shown to be enriched in autoreactive BCRs.

Another finding that favors the idea of evolution of cell populations is the upregulation of *RRAS2* (and GFP) expression in the murine Rosa26-*RRAS2* models. We still do not know the molecular mechanism that results in what appears to be a shift from approximately 2-fold overexpression of RRAS2 to a mean of 30-fold overexpression. The two clearly distinct cell populations (GFP^low^ and GFP^high^) suggest the activation or loss of a single regulator of *RRAS2* expression resulting in the shift. Whatever the mechanism, it is clear that the initial advantage provided by moderate *RRAS2* overexpression is followed by the selection of cells with the highest expression of R-RAS2 that progressively dominate the leukemic cell population with age. This shift in R-RAS2 expression is accompanied by the expression of typical leukemic markers and by stronger activation of BCR-dependent pathways. Interestingly, a time-wise dependence of *RRAS2* overexpression is also observed in human patients with full-blown CLL, with overexpression peaking (approximately 30-fold) in the oldest human patients, > 80 years old. Ideally, demonstrating this evolution and selection would require measuring *RRAS2* expression along several years in the same untreated individual patients.

In addition to CLL, *RRAS2* mRNA is found overexpressed in other hematological malignancies as well as carcinomas, including non-Hodgkin’s lymphoma, liver hepatocellular carcinoma, and squamous carcinomas of the lung, head, and cervix (PCAWG cohort, Fig. 1C). These data correlate with higher levels of R-RAS2 protein found through immunohistochemical analysis of lymphomas, squamous carcinomas of the oral cavity and esophagus [18, 19], and also lung cancer (our unpublished data). Therefore, we would expect Rosa26-*RRAS2*xSox2-Cre mice overexpressing R-RAS2 in all tissues to develop additional types of cancer. This research together with the finding of the association of *RRAS2* expression levels, and the SNP rs8570, with poorer prognosis in CLL could place *RRAS2* both as a prognosis marker in different human cancers and as a molecular target for direct inhibitors, similarly to cancers with mutations in *KRAS* (reviewed in [53]).

## Conclusions

In summary, we show that overexpression of the wild type form of a rather neglected oncogene, *RRAS2*, is behind the development of the most frequent leukemia (CLL) in the western world. Its deliberate overexpression in a mouse model provokes the development of the leukemia, thus demonstrating a cause-effect relationship. In addition, it is very frequently found overexpressed in human CLL; with higher overexpression associated with more lymphocytosis, with advanced age and with male sex, all conditions of more aggressive disease. The cause-effect relationship between *RRAS2* overexpression and CLL is reinforced by the finding that a SNP in the 3’UTR of the *RRAS2* mRNA is associated with more lymphocytosis, fewer platelets, chromosomal aberrations and with full-blown disease. Mechanistically, we find that R-RAS2 protein is physically and functionally associated to the BCR, being important for the activation of the PI3K-Akt-mTOR and other BCR-driven pathways, thus linking R-RAS2 with a previously known player in CLL cell survival and proliferation. We believe this study brings to light an important driver in human cancer which, unlike better known RAS members, does not require to bear oncogenic mutations to provoke cancer and can become itself a drug target for the treatment of CLL and probably other cancers.

## Supplementary Information

**Additional file 1: Figure S1. a,** Relative mRNA expression of *RRAS2* in different types of leukemia. Data comes from (Haferlach et al., 2010) and has been retrieved from www.oncomine.org. **b,** Schematic representation of the overexpression cassette inserted into the Rosa26 locus. **c,** Relative expression of *RRAS2* measured by RT-qPCR in different organs of Rosa26-*RRAS2*^fl/fl^xSox2-Cre (Sox2-Cre+) mice compared to that of WT C57BL/6 J Control mice using 18S as the reference gene. All expression numbers were normalized to those of liver from WT Control mice (mean = 1). Data show relative expression of *RRAS2* in the indicated organs in *n* = 3–4 8 month-old independent mice. **d,** Quantification of spleen weight from control and 6 month-old Sox2-Cre + mice. Data shown correspond to four control mice and eleven Sox2-Cre mice. Two-tailed unpaired t-test with Welch’s correction. **e,** Two-parameter flow cytometry of the expression of CD5 and IgM in B cells in the spleen of 6 month-old control and Sox2-Cre + mice. **f,** Quantification of the number of CD5 + IgM+ B cells in the spleens and bone marrow of 6 month-old control and Sox2-Cre + mice. Data correspond to triplicate measurements of one control and three Sox2-Cre mice. Unpaired t-test with Welch’s correction. **g,** Quantification of the serum IgM concentration in the blood of 35–40 wk-old control (*n* = 3) and mb1-Cre (*n* = 8) mice by ELISA. Unpaired t-test with Welch’s correction. **h,** Representative images from Giemsa stainings of blood smears of 36 wk-old control and mb1-Cre mice. **i,** Two-parameter flow cytometry of the forward scatter and CD5 expression in CD19+ cells in the blood of 16 wk-old mb1-Cre mice. The gated population represents large cells. **j,** Two-parameter flow cytometry of CD5 expression and BrdU incorporation in CD19+ cells in the blood of 16 wk-old mb1-Cre mice. **k,** Quantification of the percentage of CD19+ cells that are CD5+ blasts and of the CD19+ CD5+ cells that have incorporated BrdU.

**Additional file 2: Figure S2. a,** Flow cytometry analysis of GFP populations in 23 wk-old Rosa26-*RRAS2*^fl/fl^xSox2-Cre mouse spleen. Representative two-color contour plots of GFP^high^ and GFP^low^ populations in total B cells (CD19+), CD5+ leukemic and CD23+ follicular B cells. Bottom, representation of GFP populations in T lymphocytes (CD3+). **b,** Percentage of GFP^high^ cells in the indicated populations determined by flow cytometry. Data show means ± SEM from *n* = 8 mice (23 wk-old mice). ****p < 0.0001 (one-way ANOVA test). **c,** Western blot analysis of R-RAS2 expression of sorted GFP^low^ and GFP^high^ leukemic cells from the spleen of a 25 wk-old Rosa26-*RRAS2*^fl/fl^xSox2-Cre mouse (β-actin as loading control). **d,** Dot plot representation of GFP^low^ CD5+ leukemic B cell evolution in mb1-Cre mice over time, showing each mouse individually (*n* = 14). Data points were adjusted to a linear fit. These data were retrieved from the same mice as in Fig. 2i. **e,** Percentage of CD5+ cells in the indicated populations comparing GFP^high^ and GFP^low^ distribution. Data show means ± SEM from *n* = 4 30 wk-old mice. Two-way ANOVA test. **f,** Heatmap of RNAseq expression data showing the genes differentially regulated in wild-type, follicular B cells (*n* = 6, 12wk-old), leukemic CD19 + CD5+ B cells (*n* = 6, 54wk-old), CD19+ GFP^high^ (*n* = 2, 54wk-old) and CD19+ GFP^low^ (*n* = 2, 54wk-old) populations. Only genes significantly different between GFP^high^ GFP^low^ populations (*p* < 0.05) and with a difference of 2-fold or more were used. Gene expression is shown in normalized log2 fold change.

**Additional file 3: Figure S3. a,** Representative two-color contour plots of B cell populations in a peritoneal wash and the spleen of 12 wk-old mice according to the expression of the CD11b and CD5 markers in the CD19+ population. The blue square indicates CD11b + CD5- B1b cells in the peritoneum. Red square, the presence of CD11b + CD5+ B1a cells in control mice and leukemic cells. Quantification of CD11b + CD5+ cells is shown to the right in box and whiskers plots showing all points and median value. **p < 0.01; *** *p* < 0.001, two-tailed unpaired t-test with Welch’s correction. **b,** Representative two-color contour plots of IgM and GFP expression within the CD11b + CD5+ populations shown in a. Quantification of IgM^bright^ cells within the CD11b + CD5+ B cell population is shown to the right in box and whiskers plots showing all points and median value. **** *p* < 0.0001, two-tailed unpaired t-test with Welch’s correction.

**Additional file 4: Figure S4. a,** Representative two-color contour plots of lymphoid populations in liver and spleen from 2 wk-old mice according to the expression of CD19 and CD5 and within the CD19 + CD5+ population according to the expression of CD21, B220, CD24, CD23 and CD38 markers. **b,** Column plots show the quantification of the percentage of CD19 + CD5+ B cells in liver and spleen bearing the markers shown in a. *n* = 4 mice per group. ** *p* < 0.01 ****p < 0.0001, ns, not significant (one-way ANOVA test).

**Additional file 5: Figure S5. a,** Principal component analysis of CD19 + CD21-CD23+ follicular B cells from Rosa26-*RRAS2*xmb1-Cre mice, CD19 + CD21-CD23+ follicular B cells from WT C57BL/6 J mice and of leukemic CD19 + CD5+, GFPlow and GFPhigh cells from Rosa26-*RRAS2*xmb1-Cre mice. **b,** Ingenuity Pathway Analysis (IPA) of differentially expressed genes associated with molecular mechanisms of cancer in leukemic versus normal follicular B cells. Pink-filled symbols: upregulated genes. Green-filled: downregulated genes. Double circle: protein complex; horizontal ellipse: transcription regulator; vertical ellipse: transmembrane receptor, diamond: enzyme; trapezium: transporter; triangle: phosphatase; inverted triangle: kinase; vertical rectangle: G protein-coupled receptor; circle: other. Black arrows: direct interactions; grey/white arrows: indirect interactions. Relationship labels: A: activation; B: binding; C: causation; CO: correlation; E: expression; EC: enzyme catalysis; I: inhibition; L: molecular cleavage; LO: localization; M: biochemical modification; miT: microRNA Targeting; P: phosphorylation/dephosphorylation; PD: protein-DNA binding; PP: protein-protein binding; PR: protein-RNA binding, RB: regulation of binding; RE: reaction; T: transcription; TR: translocation; UB: ubiquitination.

**Additional file 6: Figure S6. a,** Mutations found in human cancer involving the *RRAS2* gene. Data obtained from cBioPortal (97,250 patients/100669 samples). Refseq: NM_012250. Ensembl: ENST00000256196. CCDS: CCDS7814. Uniprot: RRAS2_HUMAN. Missense mutations (green dots): 36. Truncating mutations (black dots): 6. Splice mutations (orange dots): 5. **b,** Quantification by RT-qPCR or total mouse (*Rras2*) and human (*RRAS2*) mRNA expression in purified splenic CD19+ B cells from *Rras2*(Q72L)^fl/fl^ xmb1-Cre (Q72L) mice compared to purified B CD19+ B cells from control WT C57BL/6 mice and to CD19 + CD5+ leukemic B cells from Rosa26-*RRAS2*^fl/fl^xmb1-Cre mice. Results show data obtained in triplicate normalized to the C57BL/6 control for *n* = 3 mice per group. All mice were 14 month-old. Data show means ± SEM for three mice per group. *p < 0.05; ns. Not significant (one-way ANOVA test). **c,** Left, quantification by flow cytometry of total B-cell number in spleens of 14 month-old control and Rras2(Q72L)^fl/fl^ xmb1-Cre mice. Right, two-parameter flow cytometry plot showing frequency of IgM + CD5+ cells within CD19+ splenic B cells of control and Rras2(Q72L)^fl/fl^ xmb1-Cre mice. **d,** Left, concentration of B-cells per microliter in blood of control and Rras2(Q72L)^fl/fl^ xmb1-Cre mice. Right, two-parameter flow cytometry plot showing frequency of CD19 + CD5+ cells within blood B cells of control and Rras2(Q72L)^fl/fl^ xmb1-Cre mice. **e,** Frequency of marginal zone (MZ) phenotype (CD21high, CD23low), and follicular (CD21low, CD23high) B cells within CD19+ splenic B cells of control and Rras2(Q72L)^fl/fl^ xmb1-Cre mice. **f,** Phosflow cytometry analysis of different elements from PI3K-Akt-mTOR, Raf-Erk and proximal BCR signaling pathways. Wild-type CD19+ follicular B cells, CD19 + CD5+ leukemic cells from spleens of Rosa26-*RRAS2*^fl/fl^xmb1-Cre mice and CD19+ non-leukemic B cells from Rras2(Q72L)^fl/fl^ xmb1-Cre are shown. In grey, background fluorescence of the secondary antibodies. All mice were 23 wk-old. Data show means ± SEM from three mice per group. *p < 0.05; **p < 0.01; ****p < 0.0001 (one-way ANOVA test). **g,** Phosflow cytometry analysis of different elements from PI3K-Akt-mTOR, Raf-Erk and proximal BCR signaling pathways. CD19 + CD5+ leukemic cells from 30 wk-old Rosa26-*RRAS2*^fl/fl^xmb1-Cre mice are compared with WT Control follicular (CD23^high^CD21^−^), marginal zone (MZ, CD23^−^CD21^high^), B1a (CD11b + CD5+) and B1b (CD11b + CD5^−^) spleen B cell populations. Data show means ± SEM from *n* = 3 mice per group. **p < 0.01; ***p < 0.001; ****p < 0.0001; ns, not significant (one-way ANOVA test).

**Additional file 7: Figure S7.** Ingenuity Pathway Analysis (IPA) of differentially expressed genes associated with mTOR signaling, immunological development, and G1-S checkpoint regulation in leukemic versus normal follicular B cells. Pink-filled symbols: upregulated genes. Green-filled: downregulated genes. Double circle: protein complex; horizontal ellipse: transcription regulator; vertical ellipse: transmembrane receptor, diamond: enzyme; trapezium: transporter; triangle: phosphatase; inverted triangle: kinase; vertical rectangle: G protein-coupled receptor; circle: other. Black arrows: direct interactions; grey/white arrows: indirect interactions. Relationship labels: A: activation; B: binding; C: causation; CO: correlation; E: expression; EC: enzyme catalysis; I: inhibition; L: molecular cleavage; LO: localization; M: biochemical modification; miT: microRNA Targeting; P: phosphorylation/dephosphorylation; PD: protein-DNA binding; PP: protein-protein binding; PR: protein-RNA binding, RB: regulation of binding; RE: reaction; T: transcription; TR: translocation; UB: ubiquitination.

**Additional file 8.**

**Additional file 9.**

**Additional file 10.**

**Additional file 11.**

## Data Availability

The data supporting the conclusions of this article are presented within article and its additional files.

## Abbreviations

CLL: Chronic lymphocytic leukemia
R-Ras2: RAS-related member 2
BCR: B-cell antigen receptor
3′-UTR: 3 untranslated region
SNP: Single-nucleotide polymorphism
PI3K: Phosphatidylinositol 3-kinase
mTOR: Molecular target of rapamycin
IgHV: Variable region of the immunoglobulin heavy chain
